# Transcriptomics of the Rice Blast Fungus *Magnaporthe oryzae* in Response to the Bacterial Antagonist *Lysobacter enzymogenes* Reveals Candidate Fungal Defense Response Genes

**DOI:** 10.1371/journal.pone.0076487

**Published:** 2013-10-03

**Authors:** Sandra M. Mathioni, Nrupali Patel, Bianca Riddick, James A. Sweigard, Kirk J. Czymmek, Jeffrey L. Caplan, Sridhara G. Kunjeti, Saritha Kunjeti, Vidhyavathi Raman, Bradley I. Hillman, Donald Y. Kobayashi, Nicole M. Donofrio

**Affiliations:** 1 Department of Plant and Soil Sciences, University of Delaware, Newark, Delaware, United States of America; 2 Department of Plant Biology and Pathology, Rutgers University, New Brunswick, New Jersey, United States of America; 3 DuPont Stine Haskell Research Center, Newark, Delaware, United States of America; 4 Delaware Biotechnology Institute BioImaging Center, University of Delaware, Newark, Delaware, United States of America; 5 Department of Biological Sciences, University of Delaware, Newark, Delaware, United States of America; Geisel School of Medicine at Dartmouth, United States of America

## Abstract

Plants and animals have evolved a first line of defense response to pathogens called innate or basal immunity. While basal defenses in these organisms are well studied, there is almost a complete lack of understanding of such systems in fungal species, and more specifically, how they are able to detect and mount a defense response upon pathogen attack. Hence, the goal of the present study was to understand how fungi respond to biotic stress by assessing the transcriptional profile of the rice blast pathogen, *Magnaporthe oryzae*, when challenged with the bacterial antagonist *Lysobacter enzymogenes*. Based on microscopic observations of interactions between *M. oryzae* and wild-type *L. enzymogenes* strain C3, we selected early and intermediate stages represented by time-points of 3 and 9 hours post-inoculation, respectively, to evaluate the fungal transcriptome using RNA-seq. For comparative purposes, we also challenged the fungus with *L. enzymogenes* mutant strain DCA, previously demonstrated to be devoid of antifungal activity. A comparison of transcriptional data from fungal interactions with the wild-type bacterial strain C3 and the mutant strain DCA revealed 463 fungal genes that were down-regulated during attack by C3; of these genes, 100 were also found to be up-regulated during the interaction with DCA. Functional categorization of genes in this suite included those with roles in carbohydrate metabolism, cellular transport and stress response. One gene in this suite belongs to the CFEM-domain class of fungal proteins. Another CFEM class protein called *PTH11* has been previously characterized, and we found that a deletion in this gene caused advanced lesion development by C3 compared to its growth on the wild-type fungus. We discuss the characterization of this suite of 100 genes with respect to their role in the fungal defense response.

## Introduction

Fungal species occupy diverse multispecies microbial communities within the natural environment and therefore are subjected to intra and interspecies interactions. In many cases, these interactions may result in beneficial or detrimental outcomes to an individual organism. Despite the widespread occurrence of such interactions in nature, there is a lack of understanding about how fungal species perceive and respond to interactions with other microbes.

Substantial effort has been placed on how higher organisms interact with microorganisms, with much emphasis on how they defend themselves from pathogen attack. Adaptive and innate or basal immunities represent well-established systems of study in humans, animals and plants. Unlike these systems, however, much less is known about how lower eukaryotic microorganisms such as fungi defend themselves from microbial infection. While it stands to reason that defense responses in lower eukaryotes may represent the fundamental basis of evolved defense responses in higher organisms, strong evidence is accumulating that clear differences in basic mechanisms exist. For example, as opposed to humans, animals, and plants, the fungal kingdom appears to lack the leucine-rich repeat (LRR) pattern recognition receptors, which are key players in the activation of defense responses for protection against pathogens [1,2]. It is suggested, however, that novel unrevealed classes of pattern recognition receptors are likely present in fungal genomes and function in detecting potentially harmful pathogens [2]. Investigating fungal responses to microbial antagonistic interactions is not only important for the understanding of fungal biology and physiology *per se*, but can also help to identify novel anti-fungal targets useful in developing new control methods for medical and agronomic purposes [3].

Fungi such as the well-characterized rice blast pathogen, *Magnaporthe oryzae* [4], are known to encounter a wide variety of environmental stresses that range from nutrient and temperature changes to interactions with other microbes [5]. In addition to all above-ground parts of the rice plant, *M. oryzae* is also able to colonize the roots [6] and would therefore presumably encounter, in its natural environment, a host of microbes that include other fungal species, bacteria and viruses, as well as protozoa and nematodes [7,8]. While almost nothing is known about its interactions with phyllosphere and rhizosphere microorganisms, their persistence in the environment, and the potential for constant interactions with broadly diverse microorganisms, imply fungal species such as *M. oryzae* have evolved effectively to defend themselves from biotic stresses.

*Lysobacter enzymogenes* [9] is a gram negative, soil-inhabiting bacterium with demonstrated antagonism against a broad range of microorganisms including fungi, oomycetes, nematodes, and other bacteria; thus, this bacterium has potential for high agricultural impact as a biocontrol agent for plant diseases [10]. The antagonistic activity displayed by *Lysobacter* spp. towards a diverse range of microorganisms is presumed to be based upon a number of bacterial attributes including the production of extracellular lytic enzymes such as proteases, chitinases, glucanases, lipases, and phospholipases [11,12]; the production of antibiotic compounds, such as myxin, dihydromaltophilin, and β-lactams [13]; the production of biosurfactant compounds, which are likely synthesized by some microorganisms to emulsify the hydrocarbon substrates and facilitate their transport [14]; and the involvement of the bacterial secretion systems on its host interactions [10,15]. The DCA strain of *L. enzymogenes*, mutated in the *clp* regulatory locus, lacks many of these attributes, including production and secretion of enzymes and antibiotics [15]. The genome sequence of *L. enzymogenes* was recently completed, revealing the presence of genes encoding for lytic enzymes and biosynthetic pathways for secondary metabolites predicted to contribute to antagonism toward fungal species. The genome sequence has further revealed the presence of genes encoding pathogenicity mechanisms prevalent in bacterial pathogens of animals and plants, including type III, type IV and type VI secretion systems (Kobayashi et al., unpublished). Presence of these genes, along with observations that *L. enzymogenes* interacts with and has the capacity to kill fungal cells directly [16], provides strong supportive evidence that the bacterium utilizes strategies similar to pathogens to colonize fungal hosts.

Despite the studies demonstrating various types of biological control of the rice blast fungus [17,18], the molecular interplay that occurs during these interactions is not well-defined. The lack of molecular information on microbial interspecies interactions is two-fold: first, very few physical details of the interaction itself are known, and second, very little is known about whether the fungal cells challenged by bacterial agents are able to mount an active defense in response to the attack. A recently published article reported the dual transcriptional profiling in a non-contact interaction between the soil bacterium, *Collimonas fungivorans* and *Aspergillus niger* [19]. Transcriptional profiling of the fungus revealed 53 differentially expressed genes for the two time-points tested in the study, and represents one of the few studies done to date that has performed whole genome transcriptional profiling in a bacterial-fungal interaction. Sequenced model fungi with established molecular techniques, such as *Aspergillus niger*, *A. nidulans* [20], and *M. oryzae*, are suitable for investigating how fungal species respond to antagonistic interspecies interactions and whether fungi are capable of mounting defense responses, such as basal immunity.

The goal of the present study was to investigate whole genome transcriptional changes in *M. oryzae* cells when challenged with *L. enzymogenes* wild-type strain C3 and compared with cells challenged with the non-pathogenic mutant strain DCA [21,22]. *M. oryzae* and *L. enzymogenes* transcriptome profiling experiments were performed at two time-points representing early and intermediate stages of the interaction. Using RNA-seq to access the fungal transcriptome, data from *M. oryzae* treated with the *L. enzymogenes* wild-type strain C3 were compared with data from the fungus treated with the *L. enzymogenes* mutant strain DCA. The RNA-seq transcriptional profiling of samples from the early time-point of 3 hpi (hours post-inoculation) and the intermediate time-point of 9 hpi rendered significantly different numbers of differentially expressed fungal genes between the two bacterial treatments, indicating fungal responses differ temporally and between bacterial strains during interactions. A sub-set of fungal genes that displayed an expression pattern of repression at 3 hpi during challenge with the *L. enzymogenes* wild-type strain C3, and induction during challenge with the mutant strain DCA, were identified and considered candidate genes for fungal defense response. The results are further discussed regarding gene functions and their putative roles in bacterial-fungal interactions.

## Results

### Microscopic evaluation reveals progression of interaction between *M. oryzae* and *L. enzymogenes*

**Figure 1:**
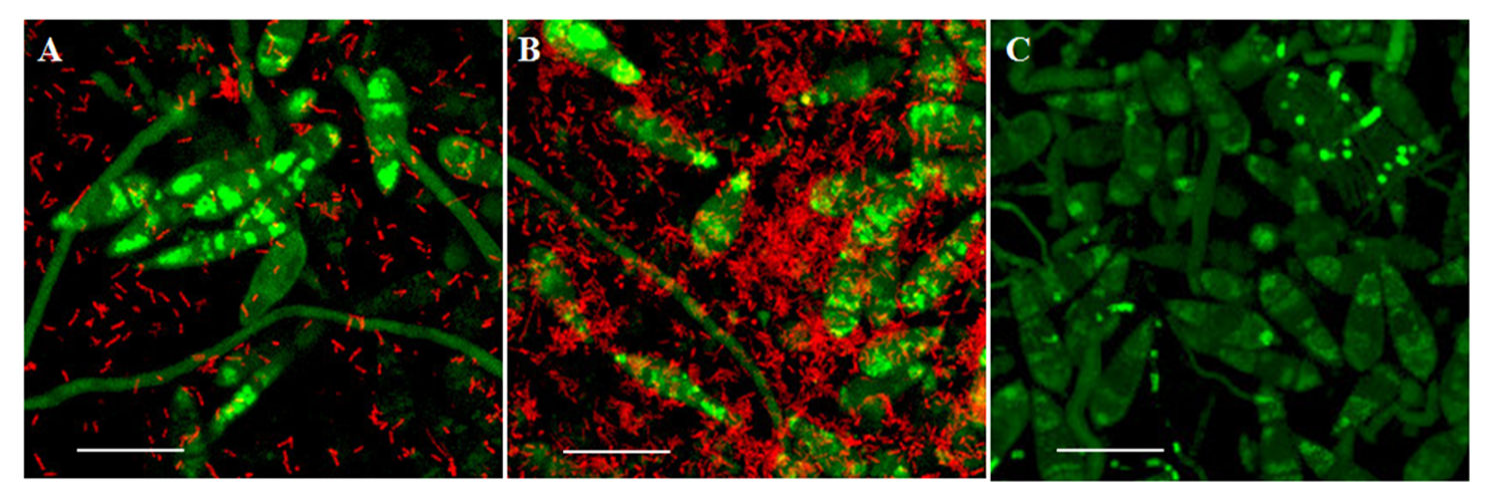
Confocal images of the interaction assay of *M. oryzae* and *L. enzymogenes* wild-type strain C3. *M. oryzae* expressing a green fluorescent protein and *L. enzymogenes* expressing a dsRed fluorescent protein at 3hpi (A) and 9 hpi (B), and a mock inoculated sample (C). The long, thin structures are hyphae, whereas the tear-drop shaped structures are conidia. The smaller red rod shapes are bacteria. *M. oryzae* conidium size ranges from 20 to 30 µm. Scale bar: 20µm.

**Figure 2:**
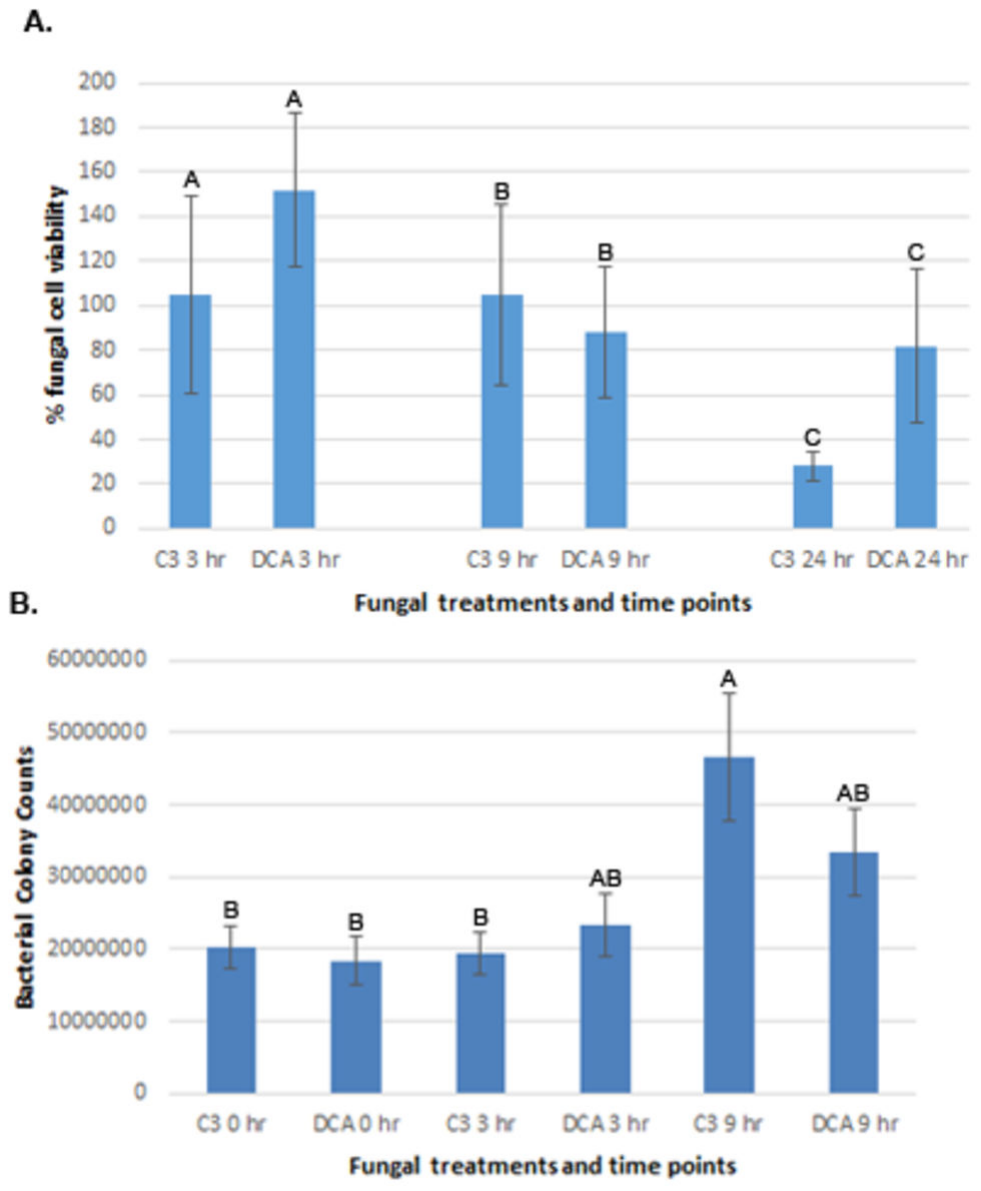
Fungal viability and bacterial load. Fungal killing assay using the MTT staining protocol to determine % viability of fungal cells, after treatment with either *Lysobacter enzymogenes* strains C3 or DCA (A). By 24 hours post-inoculation (hpi), the C3-treated sample is only 25% viable compared with untreated fungal cells, whereas the DCA-treated sample retained approximately 85% viability. Bars represent the average of 3 replicates and lines represent the standard error. Tukey-Kramer test was performed on the wild-type and mutant bacterium at each time-point (3 hr: p-value > 0.46; 9 hr: p-value > 0.76; 24 hr: p-value > 0.2). Bacterial burden assay showing no significant differences in bacterial numbers between C3 and DCA –treated samples at 0, 3 and 9 hours post-inoculation (B). Each plotted value indicates the population (cfu-colony forming units) of *L. enzymogenes* that colonized fungal cells per 100 µl inoculation. Bars represent the average of three replicates, lines represent standard error and capital letters over each bar represent lack of significance between pairs (C3 and DCA) at each time-point. Statistics were performed with the Tukey-Kramer test.

In order to investigate the interaction between *M. oryzae* and *L. enzymogenes*, we chose an *in vitro* plate assay coupled with confocal microscopy using a dsRed-expressing bacterial wild-type strain and a GFP-expressing fungal wild-type strain. This system provided both a consistent and powerful test for scrutinizing the interaction over a 24-hour period. Using this assay, we were able to determine three stages representative of early, intermediate and late stages of the interaction during co-cultures established on oatmeal agar (see Materials and Methods for details). Images taken at regular intervals over a 24-hour period revealed two time-points representing early and intermediate stages of the interaction based on the amount of bacteria in direct contact with fungal cells. Three hours post-inoculation (hpi) was chosen to represent an early stage of the interaction, when bacteria were observed to begin attaching to fungal hyphae and conidia (Figure 1A). Nine hpi was selected to represent the intermediate stage of the interaction at which time increased numbers of bacteria were observed to be in close proximity to fungal cells, and significantly larger numbers appeared attached to the hyphae and conidia compared with 3 hpi (Figure 1B). At the 9 hpi stage, the fungal hyphae were deemed to still be viable as determined both by lack of propidium iodide staining as well as retention of GFP fluorescence (data not shown). We observed that the fungus remained viable up to 12 hpi during interactions with C3. Late stages of the interaction were considered at time-points occurring after 12 hpi, when fungal cells were either observed as dead or dying. The 3 hpi mock control, treated with 1X PBS only (Figure 1C), showed no cell death as indicated by lack of propidium iodide staining and retention of GFP fluorescence during a time course of 24 hours (data not shown, except for 3 hpi in Figure 1C). Non-transformed fungal and bacterial strains showed the same pattern of interaction as the transformed strains (data not shown), indicating that neither the transformation process nor the transgene interfered with infection. Attempts at transforming the DCA mutant bacterial strain with GFP failed, however imaging with the fungus over a time-course revealed that DCA formed clumps around the hyphae, and the hyphae remained viable well past 9 hpi. We performed a second assay using the viability stain MTT in order to support the microscopy observations (Figure 2A). After inoculating the fungus with either C3 or DCA, the samples were collected and processed with MTT (see Materials and Methods for details [16]). The graph in Figure 2A shows, in percent viability compared to untreated samples (fungi alone), that up to 9 hpi fungal cells in both C3 and DCA-treated samples remained viable at levels similar to untreated fungal cells. At 24 hpi, viability of fungal cells treated with strain C3 showed a reduction to only 25% compared with untreated fungal cells, whereas viability of fungal cells treated with mutant strain DCA was measured at almost 85% compared with untreated cells. Although the Tukey-Kramer test did not show a significant difference between these two treatments at the 24-hour time-point (p-value > 0.2), there was a clear trend of less viable cells in the C3-treated samples. Since 3 and 9 hpi represented an early and an intermediate stage of the interaction at which fungal cells remained viable, these time-points were selected for RNA-seq transcriptional profiling. In order to ensure that differences in RNA-seq expression patterns in fungal cells resulted from treatment by the two different bacterial strains and not from differences in bacterial cell densities colonizing the fungal cells, bacterial populations colonizing fungal cells were determined at 0, 3 and 9 h post-inoculation. For each of the three time-points, we observed that populations of the two different bacterial strains did not differ significantly (Figure 2B).

### RNA-seq reveals *M. oryzae* transcriptome changes during time-course interactions with *L. enzymogenes*

Based on our microscopic observations, we sought to examine *M. oryzae* transcriptional profiling using RNA-seq during interactions with *L. enzymogenes* at the early stage of 3 hpi and during the intermediate stage of 9 hpi. We evaluated interactions with the *L. enzymogenes* wild-type strain C3, as well as the mutant strain DCA, which is unable to produce and secrete enzymes and antibiotics, hence unable to kill fungal cells [15]. RNA-seq was performed on two biological replicates for each bacterial treatment at each time-point, as well as on the *M. oryzae* mock-inoculated control treatment (see Figure 1C) to which each bacterial treatment was compared (see Materials and Methods for details). The RNA-seq total raw number of reads ranged from approximately 13.8 to 22.5 million (Table S1). The total number of reads that mapped to the *M. oryzae* reference list of genes (totaling 12,827 genes) ranged from approximately 8.9 to 15.5 million (Table S1).

The abundance of reads per gene was calculated and mapped to 12,203 out of the 12,827 genes present in the *M. oryzae* genome. The program Level Of gene eXpression (LOX [23]) was used to analyze differential gene expression and generate a corresponding list of fungal genes. A p-value of ≤0.01 and a 1.5 fold-change compared with the control sample were used as cut-off values to filter the data resulting in the total number of differentially expressed *M. oryzae* genes per treatment (Table 1). *M. oryzae* challenged with wild-type strain C3 had 1,048 (8.2% of total genes) and 806 (6.3%) differentially expressed genes at 3 and 9 hpi, respectively. When challenged with the mutant strain DCA, fewer genes were differentially expressed at 3 hpi (765; 6.0% of total genes), while approximately three and a half more genes were differentially expressed at 9 hpi (2,901; 22.7% of total genes) compared to the C3 challenge. In addition to the total numbers of differentially expressed genes, the two bacterial treatments also differed substantially in the number of genes induced and repressed at both time-points. At the 3 hpi time-point, there were more *M. oryzae* genes induced when challenged with mutant strain DCA (586) than with the wild-type strain C3 (167). Interestingly, more fungal genes were repressed when challenged with wild-type strain C3 (871) than when challenged with mutant strain DCA (179). At 9 hpi, quite the opposite was observed in that three and a half times more fungal genes were repressed when challenged with DCA (2183) than with C3 (598; Table 1).

**Table 1:** Listed below are all *M. oryzae* genes that were both differentially and significantly expressed during challenge with *L. enzymogenes* wild-type strain C3 or mutant DCA, for both the 3 and 9 hpi time-points.

	**3 hpi^1^**		**9 hpi^1^**	
	**C3**	**DCA**	**C3**	**DCA**
Induced				
Annotated^2^	27	227	33	121
Hypothetical	140	359	175	597
Total Genes	167	586	208	718
Repressed				
Annotated	463	47	289	1,232
Hypothetical	408	132	309	951
Total Genes	871	179	598	2,183
Total - Annotated	490	264	322	1,353
Total - Hypothetical	548	491	484	1,548
Total	1,048	765	806	2,901

^1^ Genes were analyzed with the LOX software where the cut-off for significance was p-value ≤0.01 and fold change was ± 1.5

^2^ Annotated indicates that the genes have a putative function whereas those listed as hypothetical, do not.

In the *M. oryzae* genome, only one third of the genes are functionally annotated based on their sequence homology to other species. While we recognize that differentially expressed genes annotated as “hypothetical” may have roles in the fungal defense response, we chose to further analyze only the genes with functional annotation. Among the differentially expressed fungal genes that responded to challenge by wild-type strain C3, there were 27 induced (16% of total induced) and 463 repressed (53% of total repressed) annotated genes. In contrast, treatment with the mutant strain DCA resulted in differences that were almost an order of magnitude between the number of induced and repressed annotated genes compared to the C3 treatment; 227 fungal annotated genes (39%) were induced and 47 (26%) were repressed. At the 9 hpi time-point, the number of induced and repressed genes was greater when the fungus was challenged with strain DCA than when challenged with strain C3. Challenge with C3 resulted in 33 induced (16% of total induced) and 289 repressed (48% of total repressed) annotated genes, whereas challenge with DCA resulted in 121 induced (17% of total induced) and 1,232 repressed (56% of total repressed) annotated genes. A total of 24 genes, representing 18 with annotated and 6 hypothetical functions, were selected for validation of the RNA-seq data using qRT-PCR (Table S2; Table S3). Approximately 91% of these genes could be validated, showing the same pattern as the RNA-seq result. Results ranged from 87.5 to 95.8% depending on the treatment, and indicated a robust RNA-seq dataset.

Since our RNA-seq data was obtained from sporulating fungal cultures, we wished to check whether elimination of spores would appreciably change gene expression. Fungal tissue consisting only of hyphae was collected at the 3 hpi time-point and qRT-PCR was performed for a set of genes selected from the RNA-seq analyzed data (Table S4). The expression profiles for many of the selected genes showed a similar trend to that observed in the RNA-seq (Table S5), indicating that media type and presence or absence of fungal spores did not make an appreciable difference to fungal gene changes during interaction with *L. enzymogenes.*

### Functional classification of *M. oryzae* genes differentially expressed in response to *L. enzymogenes* C3 and DCA strains

**Figure 3:**
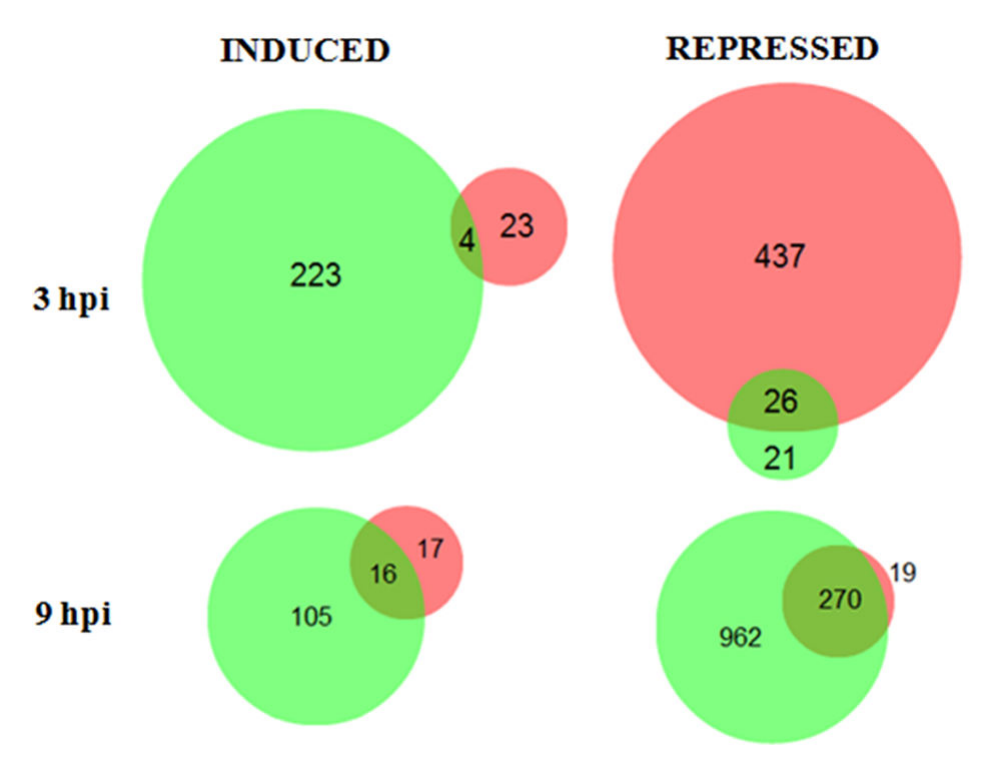
Area proportional Venn diagrams of the differentially expressed genes in *M. oryzae*. The fungus was challenged with *L. enzymogenes* wild-type strain C3 (red circles) and with mutant DCA (green circles) and the transcriptome was profiled at 3 and 9 hpi. The left and right diagrams show number of induced and repressed genes, respectively.

The genome of *L. enzymogenes* wild-type strain C3 contains a number of genes known to be involved in bacterial pathogenicity of eukaryotic hosts in other systems. These include genes encoding type III, type IV and type VI secretion systems, which function to deliver effectors from the bacterium into the host cell. Since bacterial effectors function to subvert host defense and enhance infection, we hypothesized that fungal genes that are repressed by C3 while induced by DCA represent strong candidates for fungal defense responses. In this scenario, we predict the wild-type “pathogenic” bacterial strain C3 uses effectors to repress these defense genes during attack, while the presence of the “non-pathogenic” mutant strain DCA is detected by the fungal host, thus triggering its defense response. In order to not miss any other potentially important changes though, we examined all combinations of expression patterns in conjunction with potential function of genes that were significantly differentially expressed. Area-proportional Venn diagrams were generated to represent the numbers of unique and overlapping genes between the C3- and DCA-fungal interactions for both time-points (Figure 3). At 3 hpi, 4 genes were found to be induced in both C3 and DCA treatments: the fungal cellulose binding domain-containing protein (MGG_00215.6); a laccase *TilA* (MGG_00423.6); a β-lactamase (MGG_08486.6); and a NAD/NADP octopine/nopaline dehydrogenase (MGG_10362.6). At least two of these genes (laccase and β-lactamase) are known to function in fungal response to microbial stresses [24,25,26,27]. There were 26 repressed genes that were shared between treatments with strains C3 and DCA. Among them are an alkaline protease (MGG_04733.6), a polyketide synthase (MGG_08236.6), and a serine threonine protein kinase (MGG_11636.6), which also represents genes known to be involved with microbial stress responses or signal/transduction pathways.

At 9 hpi, there were 16 fungal genes commonly induced among the two bacterial treatments, including the same β-lactamase (MGG_08486.6) induced in the 3 hpi time-point, an allantoate permease (MGG_04099.6), a glucose oxidase (MGG_07580.6), and a cytochrome P450 (MGG_08494.6). The 270 repressed fungal genes common to both bacterial treatments included two superoxide dismutases (MGG_00212.6 and MGG_07697.6) and a peroxiredoxin (MGG_02710.6). Along with having putative roles in oxidative stress management, these three genes are also up-regulated during host infection [5], indicating that candidate fungal defense response genes may also contribute to virulence.

**Figure 4:**
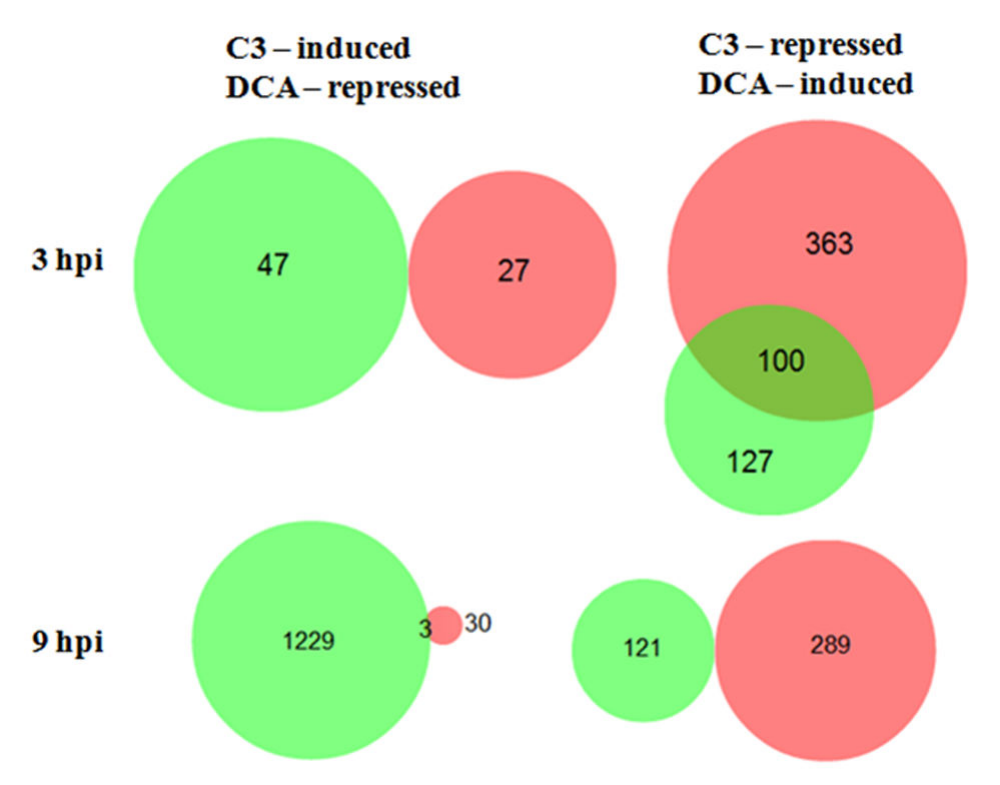
Venn diagrams reveal number of overlapping genes in *M. oryzae* challenged with *L. enzymogenes* wild-type C3 and mutant DCA. The area proportional Venn diagram shows numbers of fungal genes differentially expressed when challenged with *L. enzymogenes* wild-type strain C3 (red circles) and with mutant DCA (green circles) at 3 and 9 hpi. The induced genes by the C3 treatment were compared to the repressed genes by the DCA treatment and no overlapping genes were found at 3hpi, but 3 genes overlapped at 9 hpi. One hundred genes, which were repressed by C3 treatment and induced by DCA, overlapped at 3 hpi, whereas no overlapping genes were found at 9 hpi.

Based on our afore-mentioned hypothesis, we also wished to carefully examine *M. oryzae* genes specifically repressed during the C3 challenge and induced during DCA challenge at 3 hpi. As indicated in the Venn diagram (Figure 4), 100 genes were found to match this expression pattern (Table S6). In contrast, no genes were identified with the opposite expression pattern—induced during the C3 challenge and repressed during the DCA challenge at 3 hpi (Figure 4). At 9 hpi, only three fungal genes were found to be induced in the presence of strain C3 and repressed in the presence of strain DCA, while no genes were identified that were repressed by strain C3 and induced by strain DCA (Figure 4).

**Figure 5:**
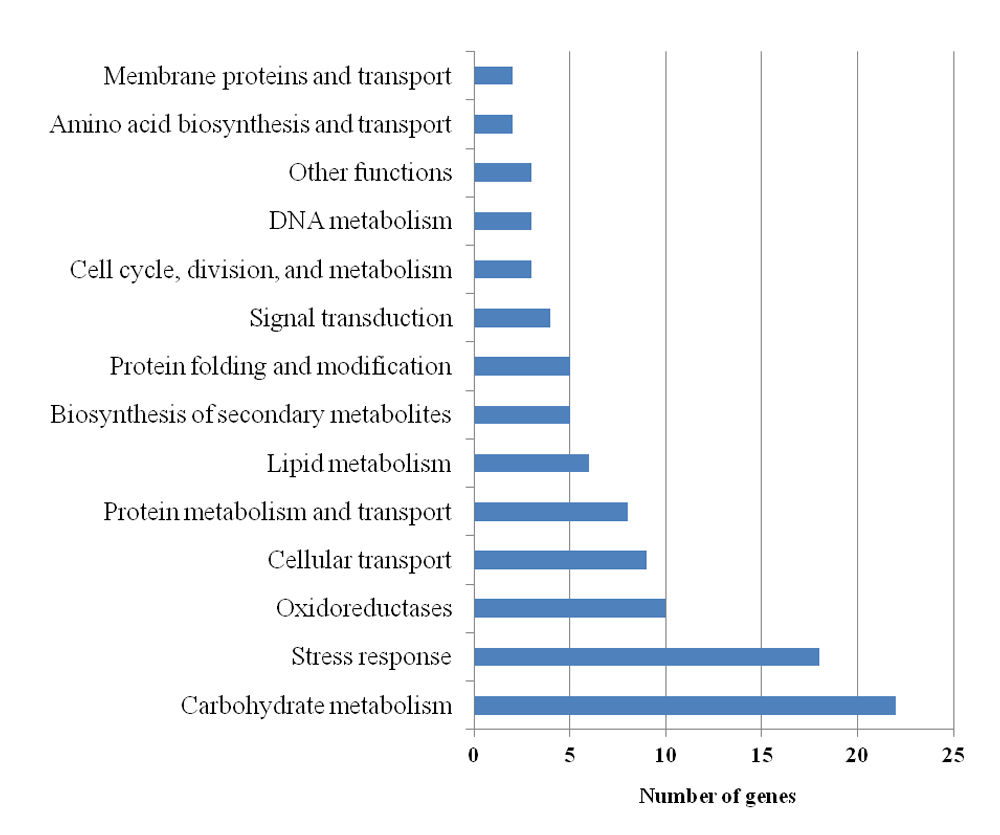
Functional categorization of *M. oryzae* genes repressed by *L. enzymogenes* wild-type C3 and induced by the mutant DCA. The graph shows functional categorization of 100 genes repressed by the wild-type bacterial strain C3 and induced by the mutant bacterial strain DCA. Numbers of genes in each functional category (y-axis) are shown across the x-axis. Genes were categorized using the Universal Protein Resource, Uniprot.

The suite of 100 differentially expressed genes from the 3 hpi time-point was categorized based on their putative function (Figure 5). The most prominent categories were carbohydrate metabolism, stress response, oxidoreductases, and cellular transport. This gene set was examined further to determine their distribution on the seven *M. oryzae* chromosomes. The majority of the 100 genes was located on chromosomes 1, 2, 3, and 6, and did not display an obvious pattern of co-localization or clustering (Figure S1).

The RNA-seq data was also investigated for genes that were either commonly induced or repressed at both 3 and 9 hpi. Challenge with C3 resulted in 2 induced and 209 repressed genes in common between the 3 and the 9 hpi time-points (Figure S2). The two induced genes are a β-lactamase (MGG_08486.6) and a 3-oxoacyl-(acyl-carrier-protein) reductase (MGG_11927.6). Only five induced and 22 repressed genes were in common between the 3 and the 9 hpi time-points for the strain DCA treatment. Among the five induced genes is one encoding for β-lactamase (MGG_08486.6), also induced at 3 hpi by both bacterial treatments.

### Predicted DNA and protein motifs, and cellular localizations are shared amongst the 100 differentially regulated genes

Groups of proteins that share structural motifs, cis-acting DNA binding motifs, and sub-cellular localization can indicate genic co-regulation [28,29]. We wished to determine whether the suite of 100 genes with similar expression patterns during the bacterial interaction shared common motifs and/or localities.

**Figure 6:**
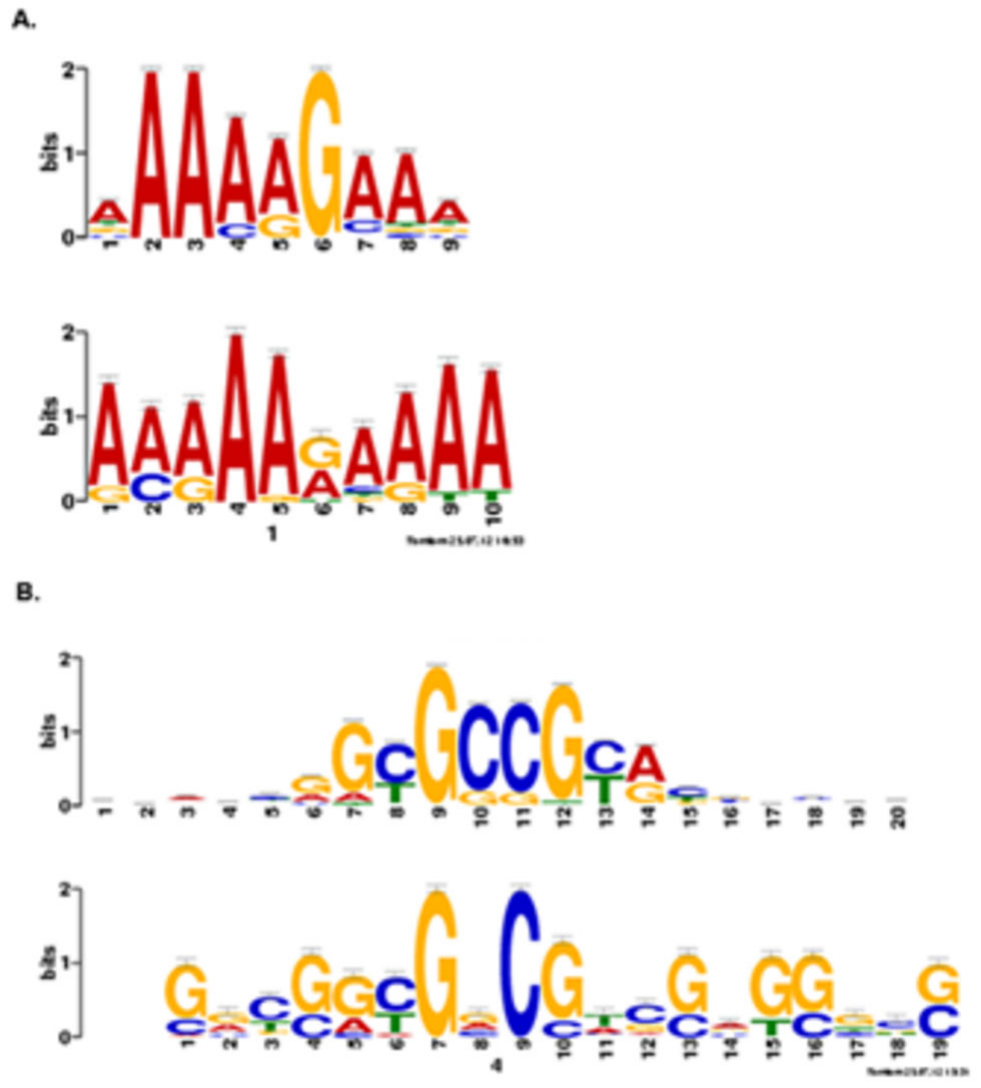
Promoter element motifs genes repressed by *L. enzymogenes* wild-type C3 and induced by mutant DCA. Promoters for the 100 fungal genes repressed by wild-type C3 and induced by mutant DCA were analyzed for common motifs using the MEME suite. Thirty-three genes had promoter elements with similarity to the binding site for the AZF1transcription factor (A) and 23 genes had promoter elements with similarity to the binding site for the STP2 transcription factor in *S. cerevisiae* (B).

A DNA promoter element analysis was performed on the genomic region 500 bp upstream of the predicted start sites for each of the 100 genes using MEME, a motif-based sequence analysis tool [30]. As a “control group” a set of 100 genes that had significant expression during treatment with strains C3 and/or DCA, but did not fit the profile of repressed by C3 and induced by DCA, were also run through the same MEME analysis. Results showed two groups of genes based on common promoter elements. The first group was composed of 33 genes (Table S7) sharing a similar promoter element. TOMTOM, a motif comparison tool within the MEME suite [30] revealed that the promoter element shared similarity (p-value ≤0.001) to the binding site for the AZF1 zinc-finger transcription factor (YOR113W) from the yeast *Saccharomyces cerevisiae* (Figure 6A). Two genes in this group, a CFEM domain-containing protein gene (MGG_07553.6) and a MSF quinate transporter (MGG_04225.6) were found to have four binding sites in their promoter regions. The *M. oryzae* genome was queried to determine whether it contained an AZF1 homolog, through a BLAST analysis of the *S. cerevisiae* sequence against *M. oryzae*. The best output resulted in an uncharacterized zinc finger C2H2-type transcription factor (MGG_03977.6), with 38% identity and 54% similarity to the yeast AZF1 at the amino acid level. Expression of this transcription factor was induced by challenge with the mutant strain DCA at 3 hpi (fold-change 0.31), while no significant change in expression was observed during challenge with the wild-type strain C3. At 9 hpi, expression was repressed during both treatments. Analysis of the control group of 100 randomly selected promoters identified a motif similar to Figure 6A (AZF1-related binding site) and with a p-value ≤0.05. However, unlike the test group, none of these genes in the control group had more than one such motif in their promoter region.

A second group composed of 23 genes (Table S8) was identified that shared another promoter element with similarity (p-value ≤0.001) to the binding site for the STP2 transcription factor (YHR006W) from *S. cerevisiae* (Figure 6B). The yeast STP2 is known to be activated in response to signals from the SPS sensor to external amino acids and also activates the expression of amino acid permease genes [31]. The gene MGG_07782.6, a member of the dehydroquinate class II enzymes, was found to have six binding sites for the STP2 transcriptional factor in its promoter region, whereas the alcohol oxidase gene (MGG_09072.6) and the translational activator GCN1 (MGG_04710.6) were each found to have five binding sites for the STP2 transcriptional factors in their promoter regions. A BLAST search analysis of the protein sequence of STP2 from *S. cerevisiae* to the *M. oryzae* genome returned the best match as a hypothetical protein (MGG_00660.6), which also has a C2H2 Zn-finger domain, and shares 29% and 42% identity and similarity at the amino acid level with STP2, respectively. MGG_00660.6 is induced by DCA (1.13) and repressed by C3 (-0.51) at 3 hpi, and repressed in both treatments -2.53 and -1.20 respectively, at 9 hpi. The control group did not share any commonalities with this second group. Only five genes shared promoter elements from both groups (AZF1 and STP2): aldehyde dehydrogenase (MGG_03900.6), lactose permease (MGG_05889.6), aldo-keto reductase (MGG_06784.6), high-affinity nickel-transporter *nixA* (MGG_05503.6), and calcium-translocating P-type ATPase (MGG_04550.6).

**Figure 7:**
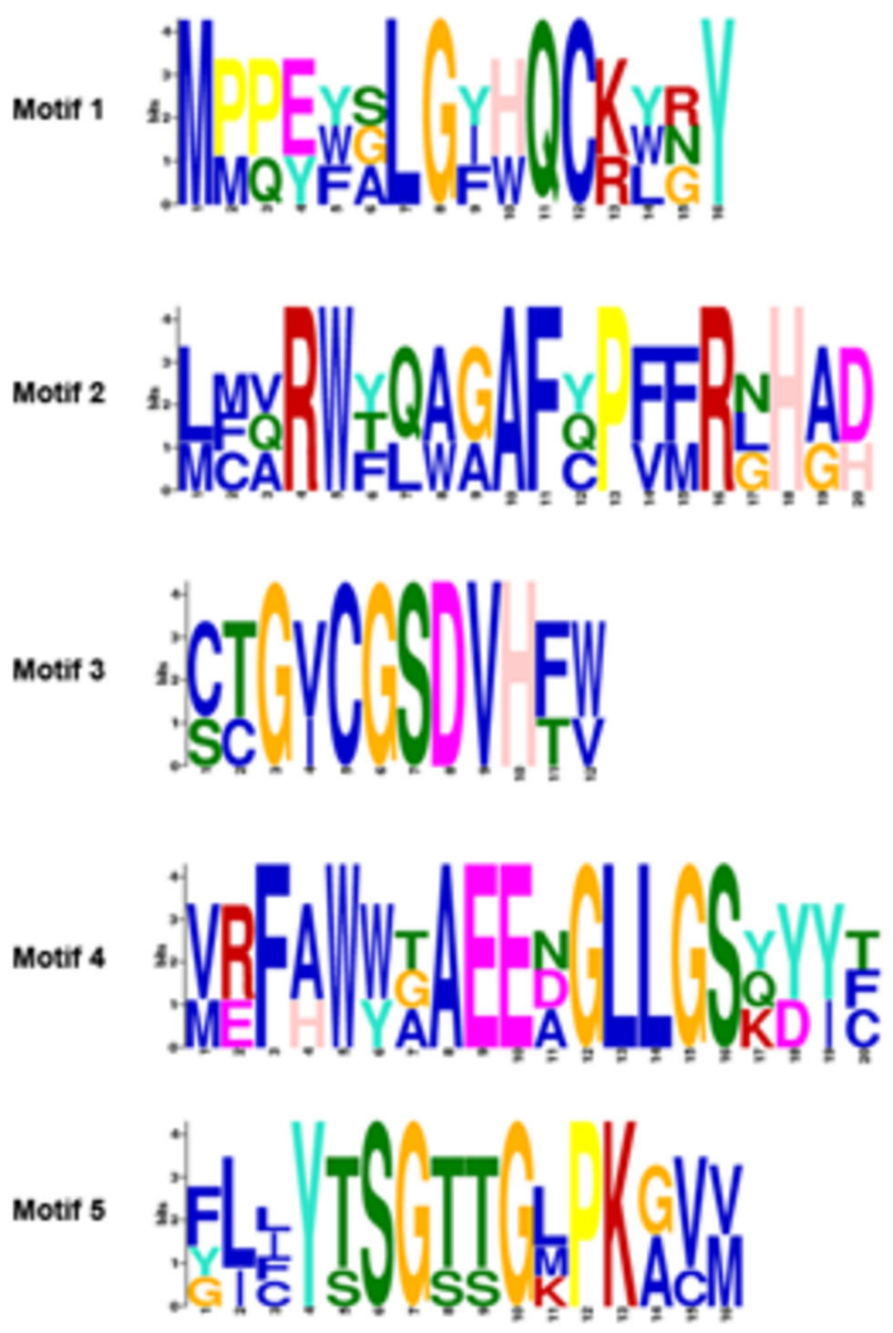
Protein motifs for genes repressed by *L. enzymogenes* wild-type C3, and induced by mutant DCA. Amino acid sequences of 100 genes were analyzed using the motif finding program MEME, revealing five significant classes. Motifs 1 and 2 were found in MGG_08623.6, MGG_10662.6, and MGG_09601.6. Motif 3 was found in MGG_09857.6, MGG_01231.6, and MGG_00220.6. Motif 4 was found in MGG_14292.6, MGG_01863.6, and MGG_06587.6. Motif 5 was found in MGG_12589.6, MGG_00689.6, MGG_03201.6, and MGG_05025.6.

Protein sequences from the 100 differentially expressed genes were queried for common motifs using MEME, and five were retrieved (Figure 7). Motifs 1 and 2 were commonly present in two glucosidases (MGG_08623.6 and MGG_10662.6) and in the xylosidase (MGG_09601.6). Motif 3 was shared by two sorbitol dehydrogenases (MGG_09857.6 and MGG_01231.6) and a NADP-dependent alcohol dehydrogenase (MGG_00220.6). Motif 4 was shared by a leupeptin-inactivating enzyme 1 (MGG_14292.6), an aminopeptidase Y (MGG_01863.6), and a leucyl aminopeptidase (MGG_06587.6). And motif 5 was shared by a 4-coumarate-CoA ligase 1 (MGG_12589.6), a propionate-CoA ligase (MGG_00689.6), an acetyl-CoA synthetase (MGG_03201.6), and a fatty acid transporter (MGG_05025.6). Studies have shown that leucyl aminopeptidase (also called leucine aminopeptidase; LAP) has a regulatory role in the immune response against herbivores in tomato [32]. *In silico* analysis for the five protein motifs revealed no matches to known functional protein motifs.

In addition to shared motifs, we also predicted the protein subcellular localization, signal peptide, and transmembrane domains for the entire gene set (Table S6). Eight proteins were predicted to have peroxisomal targeting signals, among them the peroxiredoxin (MGG_02710.6) and the peroxisomal dehydratase (MGG_04839.6). Nuclear signal peptides (NucPred≥0.6) were predicted in five proteins, including NACHT domain-containing protein (MGG_09355.6), DEAD/DEAH box helicase (MGG_07250.6), MYB DNA-binding domain-containing protein (MGG_15357.6), stress-induced-phosphoprotein 1 (MGG_08980.6), and serine/threonine protein kinase (MGG_02016.6). Transmembrane domains were predicted in 20 proteins, including several transporters, making them candidates for interactions with bacterial proteins. One gene, MGG_07553.6, was shown to have a CFEM domain, characterized by an extracellular, cysteine-rich, EGF-like motif at their N-termini [33]. MGG_07553.6 is part of an expanded family of proteins in *M. oryzae*, largely referred to as G protein-coupled receptors, or GPCRs that represent a family of membrane-bound signal transducers [33]. We pursued the family of CFEM domain-containing proteins in *M. oryzae* in more detail, as their location, function and the expression pattern of MGG_07553.6 make them possible candidates for fungal defense genes.

### CFEM domain-containing proteins are involved in fungal-bacterial interactions

As stated above, the *M. oryzae* gene MGG_07553.6 is among the 100 identified genes of interest that are differentially expressed between treatments of *L. enzymogenes* strains C3 and DCA (Table S4). This gene also belongs to the group of 33 genes sharing a promoter element similar to the binding site for the AZF1 zinc-finger transcription factor (Table S7), and is a member of the CFEM domain family of proteins. To further investigate this family as potential fungal defense candidates, we first performed a BLAST search in the *M. oryzae* genome using the MGG_07553.6 protein sequence and retrieved 40 genes with significant similarity to CFEM-domain proteins. These 40 genes differed in the number of predicted transmembrane domains, ranging from 3 to 17 (Table S9). Next, we examined their RNA-seq expression values at 3 hpi, and this revealed that thirteen family members were differentially regulated during interactions with strain C3 and mutant strain DCA; while the vast majority showed down-regulation during treatments with both bacterial strains (Table S10, yellow highlighting), two showed expression patterns of repression during challenge with strain C3 and induction with strain DCA (MGG_07553.6 and MGG_03584.6; green highlighting), and one showed the opposite pattern (MGG_12476.6; blue highlighting). Differential regulation of the same 40 CFEM genes at 9 hpi was mostly not significant (data not shown).

Because the expression patterns of MGG_07553.6 and MGG_03584.6 fit the profile of genes predicted to be involved in fungal defense response (i.e. suppressed by wild-type strain C3) we further analyzed their sequences for clues to function. A BLAST search revealed that MGG_07553.5 and MGG_03584.6 have 50% amino acid similarity to each other, and 50% and 43% similarity at the amino acid level to the CFEM family member PTH11 (MGG_05871.6), respectively. *PTH11* was previously characterized in the *M. oryzae* strain 4091-5-8 (hereafter 4091 [34]), has been shown to be involved in appressorial formation, and as predicted by its seven transmembrane domains, localizes to the cell membrane [34]. While the *PTH11* homologue in *M. oryzae* 70-15 (MGG_05871.6) did not show significant differential expression between treatments with strains C3 and DCA at 3 hpi in our study (Table S10), we nonetheless tested the interaction of *L. enzymogenes* against a *PTH11* mutant strain in the 4091genetic background ( [35]; hereafter *Δpth11*), presenting the opportunity to further characterize a CFEM domain-containing protein and its response to bacterial attack. This gene was found to be involved in sensing surface cues that lead to formation of appressoria, rarely forming these structures, resulting in decreased disease lesions [36].

**Figure 8:**
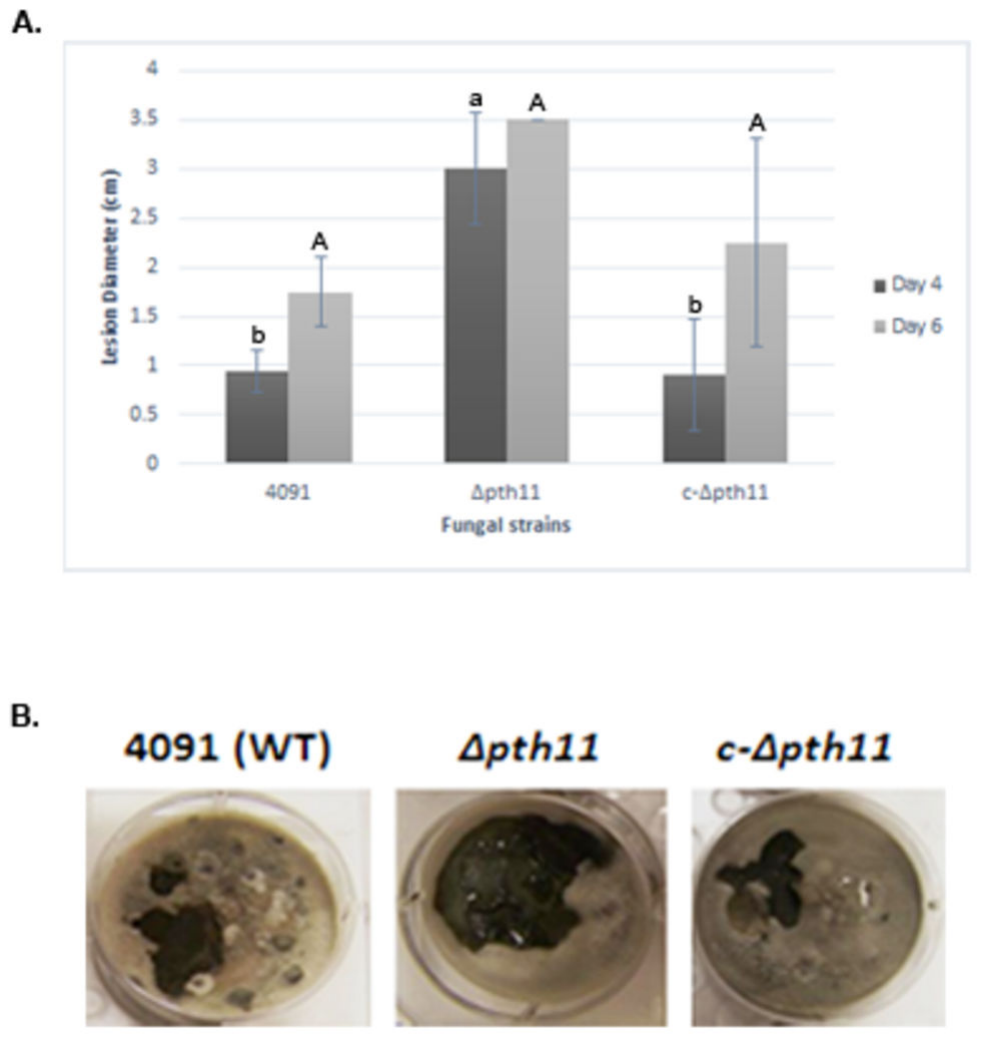
Bacterial lesions are larger on the fungal mutant *pth11* versus on the wild-type fungus. Graph shows measurements of lesion diameters caused by the wild-type bacterial strain, C3, on the wild-type fungus, 4091, the *Δpth11* mutant, and the complemented line, *c- Δpth11* (A). Bars represent the average of two biological replicates, and the lines represent error bars. Lowercase letters indicate significance at Day 4 (p-value > 0.03) and uppercase letters indicate insignificance at Day 6 (p-value > 0.14). Statistics were performed with Tukey-Kramer HSD test. Images are representative of three biological replicates. *M. oryzae* mycelia were grown on oatmeal agar for 12 days in 6-well plates (each well is 3.5 cm in diameter) and inoculated with a 40 µl drop C3 (left of center) and a 40 µl drop of DCA (right of center) (B). The image shows lesions 4 days post-inoculation. The DCA mutant bacteria never caused lesions, while the C3 wild-type strain caused large lesions on the *Δpth11* mutant.

To examine its phenotype during interactions with *L. enzymogenes*, *Δpth11* was compared with the 4091 parental strain after inoculating with bacterial cell suspensions of strains C3 and DCA (Figure 8A). Using an *in vitro* mycelial colonization assay [10], inoculation of wild-type strain C3 onto *M. oryzae* parental strain 4091 resulted in the formation of cell lysis lesions at the point of inoculation in the mycelial mat beginning at least 4 days after inoculation (dpi). By this time, lesions had spread beyond the inoculation point (Figure 8B). In contrast, inoculation of strain C3 on the mycelial mat of *M. oryzae* mutant strain *Δpth11* resulted in formation of lesions that spread more quickly, and were larger in size than those found on 4091 (Figure 8). To further verify that the enhanced lesion size resulted from the *PTH11* mutation, we inoculated a *Δpth11* complemented line (c- *Δpth11*) with C3 and DCA. As expected, DCA did not produce lesions. C3 lesions on c- *Δpth11* and 4091 did not differ, and showed some significant difference from *Δpth11* (Figure 8).

## Discussion

Little is known about the molecular interactions between fungal pathogens and bacteria, and whether or how fungi defend themselves from bacterial attack. In the present study, we took advantage of confocal microscopy and RNA-seq to study the interaction of *M. oryzae* and *L. enzymogenes*. We selected this bacterium as a model due to its demonstrated antagonism against a broad range of microorganisms and its potential for biocontrol, while the fungus was selected for its agronomic importance, fully sequenced genome and ease of genetic manipulations. Confocal microscopy proved to be very valuable for imaging the interaction, in order to select appropriate time-points in which to evaluate the *M. oryzae* transcriptome. Indeed, substantive changes in expression were observed between treatments at the two time-points, indicating the fungal host response is rapid and dynamic over a 9-hour period. Perhaps most importantly, genetically different strains of the bacterium, the wild-type strain C3 and the mutant DCA, allowed us to compare fungal gene expression profiles and identify a suite of 100 genes putatively involved in the fungal defense response.

For the specific conditions used, we observed that bacteria started to interact with hyphae and conidia as early as 1 hpi. However, we selected 3 hpi and 9 hpi, two time-points representing early and middle stages of the interaction prior to substantial fungal host cell death. These stages reflected times at which transcriptional changes were more likely to occur, since they represented points that optimized cell/cell interactions between the bacteria and fungus.

### Potential Candidate Genes in Fungal Defense

Based upon the large number of genes repressed in the fungus by *L. enzymogenes* strain C3, we formulated the hypothesis that the wild-type strain is able to negatively influence the expression of certain genes in *M. oryzae* at an early time-point, and that these genes could play a role in fungal host recognition of, and/or defense response during, interactions with microbial antagonists. We further hypothesize that the fungus is able to detect both strains, but it takes longer to detect C3 because of the bacterium’s ability to repress specific fungal genes, hence making a better infection court for itself. This situation is analogous to plant or animal-pathogen interactions, whereby basal immunity of the host species detects virulent pathogens, however the defenses are only strong enough to limit host colonization by the pathogen, not stop it all together (reviewed in 1).

At 3 hpi, there was a large number of genes that were repressed (463 genes) in the fungus when challenged with the *L. enzymogenes* wild-type strain C3 compared to the mutant strain DCA known to be devoid of antifungal activity (47 genes). Among the repressed genes were those whose annotations suggest involvement in stress and defense responses. Conversely, there were 227 fungal genes induced by DCA and only 27 induced by C3. These patterns support a hypothesis that C3 is actively repressing fungal defenses, while challenge by DCA “alerts” the fungus, inducing a defense gene set. This is similar to strategies used by bacterial pathogens of plants and animals that repress specific groups of host genes during early interactions (reviewed in 1). The 100 genes in common among the 463 (repressed by C3) and 227 (induced by DCA) were mostly involved in carbohydrate metabolism and stress responses. While their specific role during antagonistic interactions is not yet known, their expression profiles make them prime candidates for further functional characterization. This suite of 100 genes grouped into different functional categories. Among the stress response category is 2,3-dihydroxybenzoic acid decarboxylase (MGG_03793.6) and a peroxiredoxin (MGG_02710.6). The peroxiredoxin gene (MGG_02710.6) encodes an antioxidant enzyme, which in many living organisms is involved in cellular detoxification and it also is likely to play a role in circadian regulation [35,36]. 2,3-dihydroxybenzoic acid decarboxylase enzymes are involved in the conversion of 2,3-dihydroxybenzoic acid to catechol, which has antibiotic and defense functions in microorganisms and plants, respectively [37,38].

In the carbohydrate metabolism category, there are two sorbitol dehydrogenases (MGG_01231.6 and MGG_09857.6), two xylosidases (α – MGG_09601.6; β – MGG_8985.6), an α-glucosidase (MGG_10662.6), and an endoglucanase (MGG_09433.6). While it is obvious that cell wall-degrading enzymes such as xylosidase, α-glucosidase and endoglucanase might be increased in expression during plant infection [39], it is yet unclear what their role in bacterial attack might be, given the bacterial cell wall is not comprised of any of these substrates. However, there is at least precedence for repression of such genes in a study by Mela and colleagues, who demonstrated that in a non-contact co-culture assay with *C. fungivorans*, an *A. niger* endoglucanase gene (An16g06800) was repressed [19]. Another gene in this category is the conidial yellow pigment biosynthesis polyketide synthase (PKS) gene (MGG_07219.6), which is involved in the biosynthesis of secondary metabolites and also among the suite of 100 genes substantially repressed by C3 (13-fold) and induced by DCA (5-fold). In a related study, Schroeckh and colleagues showed that polyketide biosynthesis was induced during the interaction between *A. nidulans* and the bacterium *Streptomyces hygroscopicus* [20]. The gene from our study, MGG_07219.6, was also shown to increase in expression during appressorial development on an inductive surface [40]. PKS genes have demonstrated importance in the rice blast fungus; the hybrid PKS/non-ribosomal peptide synthetase ACE1 mediates an avirulent reaction in rice hosts containing the resistance gene *Pi33* [41] and PKS genes have demonstrated involvement in production of mycotoxins and host selective toxins [42,43]. This gene might produce a secondary metabolite involved in fungal protection and its deletion would reveal whether the fungus becomes hyper-susceptible to bacterial attack. On the other hand, one PKS (MGG_08236.6) showed repression in our study during both the C3 and DCA treatments, indicating that PKS genes, of which there are 23 in *M. oryzae*, could play very different roles during bacterial attack.

Our result with the *Δpth11* mutant of *M. oryzae* strongly supports the hypothesis that the fungus does indeed possess genes that defend against microbial attack; in this case, by limiting the spread and colonization of the bacterial antagonists. PTH11 is known to activate appressorial differentiation in *M. oryzae* in response to inductive surface cues and represses differentiation on poorly inductive surfaces [34]. The observation that lytic lesions created by *L. enzymogenes* wild-type C3 strain spread faster on the mycelium of fungal mutant strain *Δpth11* compared to that on the parental wild-type fungal strain indicates that the cell membrane protein may also be involved in sensing and/or signaling of the basal defense response against microbial pathogen attack. PTH11 belongs to an expanded family of CFEM domain-containing proteins in *M. oryzae* whose 40 members showed differing levels of expression during bacterial attack in our study. Curiously, the transcriptional expression of *PTH11* was largely insignificant, however based on the results with the deletion mutant, we speculate that this gene is regulated at the post-transcriptional level. Future experiments will involve testing additional cell membrane-bound genes for their protein profiles, as well as generating deletion mutants of MGG_07553.6 (identified within the suite of 100 differentially regulated genes) and other CFEM family members; we hypothesize that without this gene, the wild-type C3 bacterium will spread faster, as is the case for the *Δpth11* mutant. Genetic deletions will truly allow us to determine the roles of fungal genes in basal defense.

Four fungal genes were commonly induced by wild-type strain C3 and mutant strain DCA bacterial interactions at 3 hpi, among them the laccase gene *TilA* (MGG_00423.6) and a putative β-lactamase gene (MGG_08486.6). Fungal laccases are known for being involved in the transformation of a variety of polyphenolic compounds, which includes lignin, and are suggested to be functionally involved in fungal morphogenesis, pigmentation, and pathogenesis [44]. The interaction of fungi with other microorganisms in the soil has been observed to strongly induce laccase production [25]. Additionally, the induction of laccase was observed in *Rhizoctonia solani* only when challenged by *Pseudomonas fluorescens* strains known to produce antifungal metabolites [26]. The precise function of the *M. oryzae* laccase remains to be determined; in 2009, Chen et al. reported on the deletion of two other laccases in *M. oryzae* and found that they did not compromise pathogenicity or growth of the fungus, stating functional redundancy as a possible explanation [45]. It is possible however that laccases play a key role in fungal defense, hence genetic deletion of the *TilA* homolog in *M. oryzae* is merited. *L. enzymogenes* strain C3 is known to produce dihydromaltophilin (HSAF) – which is a tetramic acid containing macrolactam and is an antifungal compound likely acting on fungal sphingolipids [46]. Some bacteria and fungi are able to produce lactams and lactamases in order to detoxify macrolactams [27]. One possible explanation for the expression of these genes is to detoxify HSAF or related compounds, since the β-lactamase gene (MGG_08486.6) was not only induced at 3 hpi but also induced at 9 hpi. On the other hand, this gene is also differentially expressed during challenge with the mutant DCA strain, suggesting its role may be against an as yet unknown secondary metabolite.

Some genes that had significant differential expression in the RNA-seq analysis but did not pass the fold-change criterion remain potential candidates for involvement in the fungal defense response. These genes include the sphingosine N-acetyltransferase *LAG1*gene (MGG_03090.6) and the vacuolar ATP synthase (MGG_06326.6). The *LAG1* gene (MGG_03090.6), which is involved in the synthesis of ceramides, was repressed by C3 and induced by DCA. A homolog of this gene, when mutated in the fission yeast *Schizosaccharomyces pombe*, was shown to increase the sensitivity to drugs [47]. From this observation we speculate that the *M. oryzae LAG1* gene was down-regulated by C3 so the fungus would become more sensitive to the bacterial antibiotics. The vacuolar ATP synthase (MGG_06326.6) was also repressed by C3 and induced by DCA. In that same study with *S. pombe*, it was shown that loss of regulation of vacuolar ATPase also led to increased drug sensitivity [47]. This enzyme is involved in drug resistance in *S. cerevisiae* and in acidification of organelles such as endosomes and vacuoles [47,48]. The low yet significant expression of these genes and their roles in yeast during drug exposure merits further investigation into their regulation at the protein level, as well as their deletion phenotypes.

The cytoskeleton has an important role in host-pathogen interaction and for this reason is kept under tight control in the cell, which is helped in part by guanosine triphosphatases (GTPases; reviewed in 49). Interestingly, bacterial pathogens secrete protein toxins, through the type III and IV secretion systems, that can affect the activity of host GTPases by mimicking endogenous regulators of the GTPase cycle [49]. Among the 463 repressed genes in *M. oryzae* by C3, there is an ARF (MGG_01472.6) and a RAN (MGG_01248.6) GTPase-activating protein. From this observation, it is possible that C3 secretes toxins that target GTPases and the cytoskeleton, as observed in plant-pathogen interactions [50], but further investigation is needed.

### Co-Expressed Genes Share Promoter Binding Sites

As co-expressed genes are more likely to contain shared transcription factor binding sites (TFBS) [51], we searched for TFBS among the 100 differentially expressed genes by C3 (repressed) and DCA (induced). The binding site for AZF1 zinc-finger transcription factor was found to be shared by 33 of the 100 differentially expressed genes. This transcription factor is involved in the transcriptional induction of CLN3, which is involved in cell cycle progression in yeast, and is also involved in the response to glucose [52]. The other shared binding site among 23 genes is for the STP2 transcription factor, which is activated by proteolytic processing in response to signals from the SPS sensor system for external amino acids and also activates transcription of amino acid permease genes in *S. cerevisiae* [31]. *M. oryzae* has a homolog to each transcription factor, sharing 54% amino acid similarity with AZF1, and 42% with STP2. An interesting experiment would be to delete these two transcription factors from the fungus and determine whether the entire suite of 33 and 23 repressed genes, respectively, becomes altered in its expression pattern during interactions with the bacterium, and whether there is a phenotypic effect on the bacterial interaction. Should their deletions have an effect on the fungal-bacterial interaction, determination of the full set of targets of these transcription factors may help to decipher the fungal basal defense pathway [53].

### Overlapping roles in fungal defense and virulence?

We took advantage of the availability of *M. oryzae* transcriptome during *in planta* and *in vitro* stress conditions, which we have published previously [5], and mined the data for the 100 genes repressed by wild-type C3 and induced by mutant DCA. We observed that seven genes which were induced in all stress conditions tested in the previous microarray experiment (temperature up-shift, oxidative stress with Paraquat, minimal medium, carbon starved, and nitrogen starved and during invasive growth in rice and barley at 72 hpi) were among the 100 genes repressed by wild-type C3 and induced by mutant DCA. Those seven genes are aminopeptidase Y (MGG_01863.6), triosephosphate isomerase 2 (MGG_03094.6), pisatin demethylase (MGG_04404.6), aldo-keto reductase (MGG_06784.6), β-xylosidase (MGG_08985.6), endoglucanase (MGG_09433.6), and n-acetyltransferase ats1 (MGG_09867.6). These genes could be considered part of a general protective mechanism against stress in the fungus, and genetic deletions will elucidate their specific roles.

## Conclusions

The robust gene expression dataset of *M. oryzae* challenged with the putative biocontrol bacterium *L. enzymogenes* wild-type strain C3 and mutant DCA generated in this study contributes significantly to a field still in its infancy. Our data has provided numerous hypotheses on whether and how fungi defend themselves from antagonistic bacterial interactions or, looking at the same question in a different way, how bacteria with biocontrol potential make better infection courts for themselves. Further, the expression patterns we noted during challenge with C3 compared to DCA, lends support to our hypothesis that this bacterial-fungal interaction has similar attributes to bacterial-plant or bacterial-human interactions; the wild-type bacterium represses fungal genes and successfully infects, while the mutant bacterium induces fungal genes and is unable to infect. The advantage of having both the wild-type and mutant strains of the bacterium, the latter being deficient in the production of lytic enzymes and antibiotics, is that the effects of single traits in the interaction can be better defined. The fungal genes that are responsive to general characteristics of the bacteria, which could potentially be considered MAMP-like molecules, could be identified by looking at genes commonly induced or repressed by C3 and DCA. Alternatively, the genes which are responsive to specific secreted components of the bacteria can be identified by looking at genes that are uniquely differentially expressed by challenge with C3 or DCA.

## Materials and Methods

### Strains and growth conditions

*Magnaporthe oryzae* strain 70-15 was used in all experiments, unless otherwise mentioned. Conidial filter paper stocks stored at -20°C were used to initiate fungal growth. The fungus was grown on oatmeal-agar medium (50 g/L oatmeal, 15 g/L agar) for 10 days, under continuous light at 25°C for conidia production. Fifteen percent glycerol stocks of *Lysobacter enzymogenes* wild-type strain C3 and the mutant strain DCA, stored at -80°C, were used to initiate bacterial cultures. Bacteria were grown in Luria Broth (LB; cat# L3522) and Luria Agar (LA; cat# L3147) medium (Sigma Chemical, St. Louis, MO) at 30°C for all experiments. The liquid cultures were overnight grown in a shaker at 30°C at 200 rpm.

### Confocal microscopy

Confocal images were taken with the Zeiss 780 upright confocal microscope housed at the BioImaging Center in the Delaware Biotechnology Institute (bioimaging.dbi.udel.edu). *M. oryzae* strain 70-15 was transformed with Zs-Green fluorescent protein [54] and *L. enzymogenes* wild-type strain C3 was transformed with dsRed tag fluorescent protein (Patel and Kobayashi, unpublished data) and were used in confocal live cell imaging to determine the two time-points representative of an early and a middle stage of interaction. Fungal cultures were grown in oatmeal plates (plate diameter of 33 mm) for 10 days and bacterial suspension of approximately 1x10^7^ cfu/mL was added to the plate. For more details on how to prepare the bacterial suspension see next session. A water immersion objective (40X) was immersed in the suspension and the fungal-bacterial interaction was imaged for 24 hours. The image sequences were analyzed with Volocity 5.0 software (Improvision, Lexington, MA).

### *M. oryzae*-*L. enzymogenes* interactions

The cultures of the *L. enzymogenes* wild-type strain C3 and mutant DCA were grown overnight in LB medium at 200 rpm at 30°C. The cultures were centrifuged at 7,000 rpm for 10 min at 4°C. The supernatant was discarded and the cultures were rinsed with phosphate buffer saline (1X PBS; Fisher Scientific, Pittsburgh, PA; cat# BP399-500). The washing step was performed for a total of two times. A spectrophotometer was used to measure the optical density (OD) at 600 nm and the cultures were re-suspended to a concentration of 1x10^7^ cfu/mL. *M. oryzae* grown on 6-well plates containing oatmeal medium (plate diameter of 33 mm) for 10 days was inoculated with 6 mL of the bacterial culture for the C3 and DCA mutant treatments. The plates were placed in a dark incubator at 25°C until each time-point was reached (3 hours – early time-point and 9 hours – middle time-point, the two time-points resulting from the confocal time-course experiment). The bacterial culture was removed and a cork borer (#6, 12 mm diameter) was used to remove and discard the central zone of the fungal culture. Then, the remaining fungal tissue was collected and immediately frozen in liquid nitrogen. Samples were stored at -80°C until RNA extraction was performed.

To evaluate bacterial burden, populations of *L. enzymogenes* colonizing fungal cells were determined at 0, 3 and 9 hours using an interaction assay similar to that described above. The only modification involved the bacterial inoculum, which consisted of spotting a volume of 100 µl directly onto mycelia. At each time-point, remaining aqueous phase of bacterial inoculum was removed and discarded. Fungal mycelia was then extracted and ground in 1 ml PBS using a mortar and pestle, before dilution plating onto 10% TSA to determine cfu/inoculation.

Viability staining using MTT was performed essentially as described [16]. Briefly, *M. oryzae* mycelia were grown in liquid complete media (10 gm sucrose, 6 gm yeast extract, 6 gm casamino acids, 1 ml *A. nidulans* trace elements per 1 L) for 14 days. After rinsing with sterile buffer, mycelia were partitioned into 0.2-0.4 g fragments and placed into 50 ml beakers. *L. enzymogenes* inoculum consisted of cells from overnight cultures that were washed in sterile buffer and re-suspended to a density of 5 x 10^8^ cfu/ml. Interactions were initiated when 3 ml of bacterial inoculum or sterile buffer were placed into beakers containing mycelia.

### *M. oryzae*-*L. enzymogenes* interaction experiment in CM

The cultures of the *L. enzymogenes* wild-type strain C3 and the mutant DCA were prepared as previously described and were re-suspended to a concentration of 1x10^7^ cfu/mL. *M. oryzae* was grown on six-well plates (well diameter of 33 mm) on complete medium (for 5 days) and was inoculated with 6 mL of the bacterial culture. A mock sample inoculated with only PBS was used as control. The plates were placed into a dark incubator at 25°C for 3 hours. The bacterial culture was removed and a cork borer (#6, 12 mm diameter) was used to remove and discard the central zone of the fungal culture. Then, the fungal tissue was collected and immediately frozen in liquid nitrogen. Samples were stored at -80°C until RNA extraction was performed.

### RNA extraction

Total RNA extraction was performed using Trizol reagent (Sigma Chemical, St. Louis, MO) following manufacturer’s instructions. Briefly, fungal samples stored at -80 °C were ground to a fine powder in liquid nitrogen, placed in Trizol, and the final pellet was re-suspended in 50 µL of autoclaved nuclease free water (Qiagen Sciences, Valencia, CA; cat. #129115). RNA was extracted from four biological replicates, which each replicate consisting of a poll of three technical replicates. Isolated RNA was purified using the RNeasy Plant Mini Kit (Qiagen Sciences, Valencia, CA) and integrity and concentration was assessed by using agarose gel and the ND-1000 NanoDrop spectrophotometer (NanoDrop Technologies, Wilmington, DE), respectively. A total of 1 µg of RNA from each of two biological replicates was pooled for RNA-seq library preparation.

### RNA-seq cDNA library preparation and sequencing

cDNA library preparation was performed according to manufacturer’s instructions for the TruSeq RNA Sample Prep Kit (www.illumina.com). Using TruSeq allowed us to enrich for fungal (eukaryotic) RNA and avoid bacterial (prokaryotic) RNA, in order to obtain a single transcriptome for sequencing. Briefly, the poly-A containing mRNAs were purified by using poly-T oligo-attached magnetic beads and then fragmented. The first and second cDNA strands were synthesized, end repaired, and adaptors were ligated after adenylation at the 3’-ends. DNA fragments containing adaptors on both ends were selectively enriched by PCR amplification. The indexed Illumina SBS libraries were pooled into three different sample sets (each in a flow cell) for sequencing. The Illumina SBS libraries were validated by qPCR to determine clustering concentration, and for fragment size using the Agilent High-Sensitivity DNA chip on an Agilent Technologies 2100 Bioanalyzer. The samples were clustered and sequenced on an Illumina HiSeq 2000 sequencing system in the Sequencing and Genotyping Center at Delaware Biotechnology Institute in the University of Delaware. The sequencing run was a 50-cycle single-read run, followed by a 7-cycle index read. Primary analysis and quality filtering of the Illumina HiSeq data was performed using the default parameters.

### RNA-seq data analysis

The RNA-seq reads were mapped to the *M. oryzae* reference list of genes by using Bowtie [55]. The reference list of genes containing 12,827 genes was downloaded from the Broad Institute (*Magnaporthe* comparative Sequencing Project, Broad Institute of Harvard and MIT, http://www.broadinstitute.org). Bowtie alignment was performed considering the best alignment with zero mismatches. The abundance of reads per gene was calculated by using a custom Perl script which used the Bowtie output file as its input file, which had the data for each individual biological replicate separately. Then, the data were analyzed for differential gene expression using LOX (Level Of eXpression; [23]). LOX employs Markov Chain Monte Carlo to estimate the level of expression and integrates sequence count tallies that are normalized by total expressed sequence count to provide expression levels for each gene relative to all treatments as well as Bayesian credible intervals. Area-proportional Venn diagrams were generated by using the BioVenn software (http://www.cmbi.ru.nl/cdd/biovenn/index.php [56]). All the genes with p-value ≤0.01 were functionally categorized using the Universal Protein Resource (Uniprot [57,58]).

### NCBI-GEO Accession Number

RNA-seq data has been deposited in the NCBI GEO database (http://www.ncbi.nlm.nih.gov/projects/geo/) and can be found under the accession number GSE43648.

### RNA-seq validation

Twenty-four genes were selected for validation by quantitative reverse transcription-polymerase chain reaction (qRT-PCR). First-strand cDNA was synthesized from total RNA using the GoScript Reverse Transcription System (Promega Corporation, Madison, WI). qRT-PCR was performed using the Real Master Mix SYBR ROX (5 PRIME, Gaithersburg, MD; cat. # 2200800) for SYBR Green fluorescence detection on a Realplex2 Mastercycler (Eppendorf, Westbury, NY). All qRT-PCR primers were tested with RT-PCR before their use. The qRT-PCR reactions were performed in a final volume of 20 µL containing 10 µL of 2.5x MasterMix, 0.06 µL of 100 µM of each forward and reverse primers, and 1 µL of cDNA. The reactions occurred at 95 °C for 2 min, followed by 40 cycles of 95 °C for 15 sec, 58 °C for 20 sec, and 72 °C for 25 sec, followed by melting curve analysis. Relative expression levels were determined by the 2^-ΔΔCt^ method based on three technical replicates per sample and using glyceraldehyde 3-phosphate dehydrogenase (GAPDH; MGG_01084.6) as the endogenous control. All qRT-PCR reactions were repeated at least twice with similar results. The sequences of qRT-PCR primers are listed in Table S2. The expression level of a specific *M. oryzae* gene was considered as validated when the expression level obtained with qRT-PCR matched the direction of the expression level observed in RNA-seq (either up or down). The percentage of matched genes relative to the total number of tested genes was calculated for each treatment. Finally, an averaged percentage of validation was calculated for all treatments.

### *M. oryzae* PTH11- *L. enzymogenes* interaction experiment

*Magnaporthe oryzae* strain 4091-5-8 was used for this experiment because it is the background of the PTH11 mutant, which was kindly provided by J. A. Sweigard. The fungus was grown in six-well plates (well diameter of 33 mm) on oatmeal-agar medium (50 g/L oatmeal, 15 g/L agar) for 11 days, under continuous light at 25°C. *Lysobacter enzymogenes* wild-type strain C3 and the mutant strain DCA were grown on Tryptic Soy Agar medium (cat # 22091 Sigma Chemical, St. Louis, MO) at 30°C for 24 hours. Liquid cultures were then started the following day in Luria broth (cat #L3522 Sigma Chemical, St. Louis, MO) and grown overnight in a dark shaker at 30°C at 150 rpm. Bacterial cultures were centrifuged in a Thermo, Fisher swinging bucket rotor in 50 ml conical tubes at 2,500 rpm for 10 minutes. Supernatant was poured out and washed once in 1X PBS buffer (cat # BP399 Fisher Scientific, Pittsburgh, PA). Cells were centrifuged as above, PBS was poured out and cells were then re-suspended in 5 ml of 1X PBS and measured with a spectrophotometer. Cells were diluted to an OD of approximately 1x10^7^ cfu/mL. Fungal plates were inoculated with 35 µl of bacterial suspension in the following way: top rows of each plate were inoculated with the wild-type strain C3 and bottom row with the mutant strain DCA as a control. Bacterial droplets were placed on the left-hand side of each well, and 1X PBS buffer was placed on the right-hand side. Plates were kept in a low-light growth chamber at 25°C. Bacterial lesions in the fungal mat were recorded starting 2 days after inoculation, and measured and photographed every two days after for approximately 8 days.

### Bioinformatic tools for promoter element and protein analyses

The promoter element analyses were performed in the 500 bp of the promoter region upstream of the predicted start site each of the 100 genes using p-value ≤0.001. Protein sequences from the 100 differentially expressed genes were queried for common motifs using p-value ≤0.001. Additionally, 100 genes were selected that did not share this particular profile (repressed by C3 and induced by DCA), but nonetheless had significant expression in response to bacterial attack, to serve as the “control” group. The MEME suite [30] was used for searching for promoter elements and for protein motifs. All the following tools were used and default parameters were used: protein subcellular localization using TargetP [59] and WoLF PSORT [60,61]; prediction of signal peptides in proteins using PrediSi [62]; prediction of Golgi localized transmembrane proteins using Golgi Predictor [63]; potential for GPI lipid modification sites in proteins by big-PI Fungal Predictor [64]; nuclear localization of proteins using NucPred [65]; prediction of peroxisomal targeting signal in proteins using PTS1 Predictor [66]; prediction of mitochondrial targeting sequence by MitoProt [67]; prediction of transmembrane helices in proteins [68].

## Supporting Information

Figure S1 **Chromosomal distribution of fungal genes repressed by *L. enzymogenes* wild-type C3 and induced by mutant DCA.** One hundred genes repressed by C3 and induced by DCA localized to all seven fungal chromosomes, with no detectable particular distribution pattern.
(TIF)

Figure S2 **Overlapping genes between two time-points in *M. oryzae* challenged with *L. enzymogenes* wild-type C3 and mutant DCA.** Only two genes were commonly induced in the C3 treatment at 3 (red circles) and 9 hpi (green circles), while 209 genes were commonly repressed in the C3 treatment at 3 and 9 hpi. Five genes were induced and 22 were commonly repressed in the DCA treatment at 3 and 9 hpi.
(TIF)

Table S1 **Total number of raw and mapped reads for *M. oryzae* RNA-seq libraries.**
(DOCX)

Table S2 **Primers used in this study.**
(DOCX)

Table S3 **qRT-PCR results for validation of *M. oryzae*-*L. enzymogenes* RNA-Seq** **results**.
(DOCX)

Table S4 **One hundred *M. oryzae* annotated genes repressed by *L. enzymogenes* wild-type strain C3 and induced by mutant DCA at 3 hpi.**
(DOCX)

Table S5 **Results of qRT-PCR performed on *M. oryzae* mycelial samples challenged with *L. enzymogenes. M. oryzae* mycelia (no spores) were challenged with *L. enzymogenes* wild-type strain C3 and mutant DCA and the expression level of nineteen genes was examined by qRT-PCR and compared to the RNA-seq results.**
(DOCX)

Table S6 **Subcellular localization, signal peptide, and transmembrane domains for the 100 differentially expressed genes.**
(XLSX)

Table S7 **Thirty-three genes that contain the promoter element for AZF1 from *S. cerevisiae*.**
(DOCX)

Table S8 **Twenty-three genes that contain the promoter element for STP2 from *S. cerevisiae*.**
(DOCX)

Table S9 **CFEM domain-containing genes in *M. oryzae*, gene expression during bacterial challenge and number of transmembrane domains.**
(XLSX)
