# Supplementary figures and images for: Transcriptomics of the Rice Blast Fungus Magnaporthe oryzae in Response to the Bacterial Antagonist Lysobacter enzymogenes Reveals Candidate Fungal Defense Response Genes
Source: PLoS One. 2013 Oct 3;8(10):e76487. doi: 10.1371/journal.pone.0076487 (PMC3789685; doi:10.1371/journal.pone.0076487)

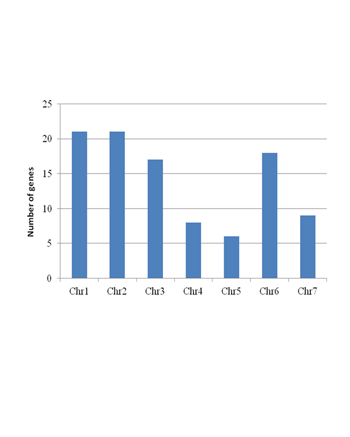

Supplement: Figure S1 — Chromosomal distribution of fungal genes repressed by L. enzymogenes wild-type C3 and induced by mutant DCA. One hundred genes repressed by C3 and induced by DCA localized to all seven fungal chromosomes, with no detectable particular distribution pattern. (TIF) [file pone.0076487.s001.tif]

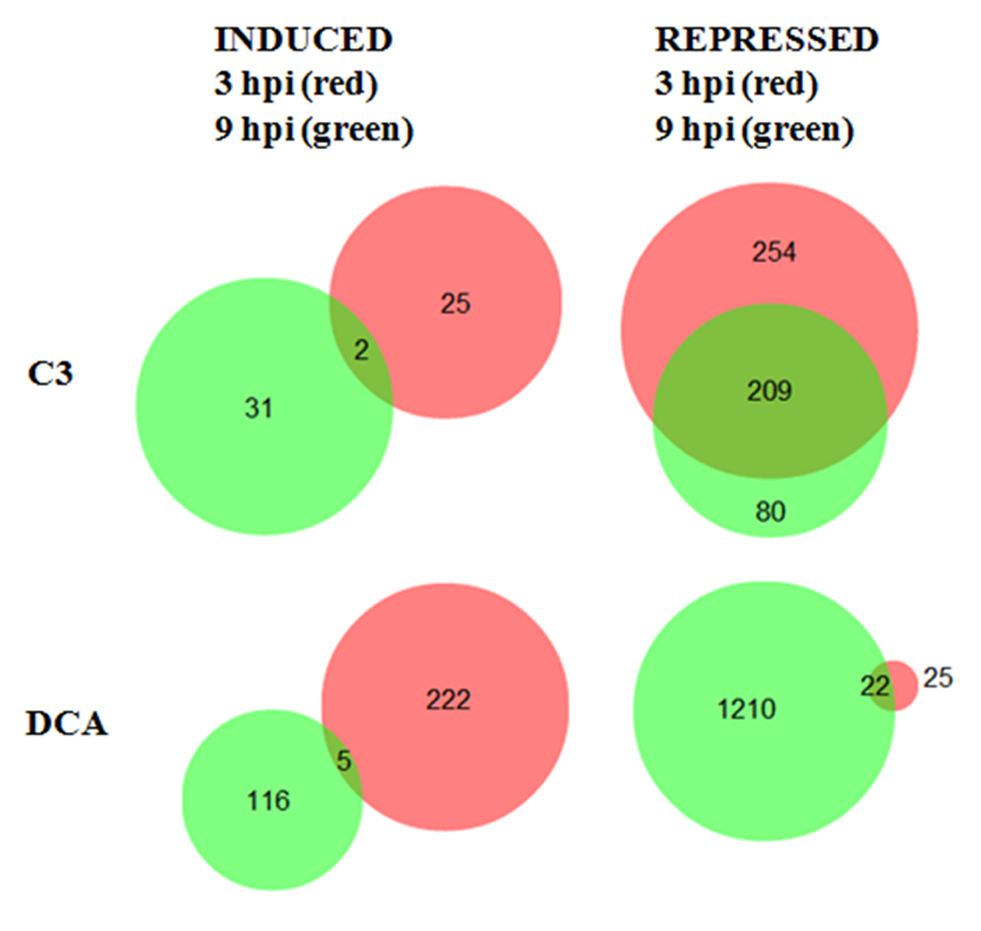

Supplement: Figure S2 — Overlapping genes between two time-points in M. oryzae challenged with L. enzymogenes wild-type C3 and mutant DCA. Only two genes were commonly induced in the C3 treatment at 3 (red circles) and 9 hpi (green circles), while 209 genes were commonly repressed in the C3 treatment at 3 and 9 hpi. Five genes were induced and 22 were commonly repressed in the DCA treatment at 3 and 9 hpi. (TIF) [file pone.0076487.s002.tif]
