# Supplementary material for: Transcriptomics of the Rice Blast Fungus Magnaporthe oryzae in Response to the Bacterial Antagonist Lysobacter enzymogenes Reveals Candidate Fungal Defense Response Genes
Source: PLoS One. 2013 Oct 3;8(10):e76487. doi: 10.1371/journal.pone.0076487 (PMC3789685; doi:10.1371/journal.pone.0076487)
Supplement: Table S1 — Total number of raw and mapped reads for M. oryzae RNA-seq libraries. (DOCX) [file pone.0076487.s003.docx]

**Table S1.** Total number of raw and mapped reads for *M. oryzae* RNA-seq libraries.

| **Library** | **Total raw reads** | **Total mapped reads** |
| --- | --- | --- |
| ^1^CON_3hpi_rep1 | 18,649.981 | 12,086.579 (65%) |
| CON_3hpi_rep2 | 19,055.536 | 12,471.900 (65%) |
| CON_9hpi_rep1 | 13,757.547 | 8,957.011 (65%) |
| CON_9hpi_rep2 | 22,461.247 | 14,539.579 (65%) |
| ^2^C3_3hpi_rep1 | 18,652.525 | 12,036.300 (65%) |
| C3_3hpi_rep2 | 18,710.154 | 12,076.093 (65%) |
| C3_9hpi_rep1 | 15,310.373 | 9,739.718 (64%) |
| C3_9hpi_rep2 | 18,920.877 | 12,165.379 (64%) |
| ^3^DCA_3hpi_rep1 | 16,925.682 | 10,848.082 (64%) |
| DCA_3hpi_rep2 | 18,776.691 | 10,363.864 (55%) |
| DCA_9hpi_rep1 | 22,143.397 | 13,940.187 (63%) |
| DCA_9hpi_rep2 | 14,566.818 | 9,233.831 (63%) |

^1^CON is the control with 1X PBS

^2^C3 is the wildtype

^3^DCA is the clp mutant
