# Supplementary material for: Transcriptomics of the Rice Blast Fungus Magnaporthe oryzae in Response to the Bacterial Antagonist Lysobacter enzymogenes Reveals Candidate Fungal Defense Response Genes
Source: PLoS One. 2013 Oct 3;8(10):e76487. doi: 10.1371/journal.pone.0076487 (PMC3789685; doi:10.1371/journal.pone.0076487)
Supplement: Table S2 — Primers used in this study. (DOCX) [file pone.0076487.s004.docx]

**Table S2**. Primers used in this study.

| **Name** | **Sequence** |
| --- | --- |
| MGG_03090-F | CAGCTGCGGCGCACGATC |
| MGG_03090-R | CAGACATGAGTGAGGGCTAGGAGC |
| MGG_05189-F | CGGCCTGGCTACAAGCATCG |
| MGG_05189-R | GGAGGACAAGAGGCAGTACGCAG |
| MGG_10668-F | GCTCATGGACGGGCTGGCTTATG |
| MGG_10668-R | GAGTGAAGGCGATACTGGTGGCG |
| MGG_00153-F | GGCCTGACCTGGCTAAGAAGCTG |
| MGG_00153-R | CATCCTCGTCGCCATGCTCG |
| MGG_05499-F | GGCAGTCTCACAGCTGGCAC |
| MGG_05499-R | GCTGCCGTTGACGTGCTCG |
| MGG_07580-F | GTCGACTGGAACTACCGCACC |
| MGG_07580-R | CTCCAGCGCGTCGATCTCG |
| MGG_01236-F | GAGCAGCAGCAGCGAGTCCTC |
| MGG_01236-R | CTAACTCCGCAGGCGTTGGC |
| MGG_06035-F | CCTTAAGGACACCAGCAAGCCTG |
| MGG_06035-R | CACGCTGAACACCGATAGCCG |
| MGG_02710-F | CGACGGTGTCTCGTTCACCTACG |
| MGG_02710-R | GTGCTGCTCCTGGCACGTAG |
| MGG_01081-F | CTGCTCGGGGAGGATGAGAG |
| MGG_01081-R | GCATTTGTGGTTGCGGTGGC |
| MGG_04404-F | CAGCCAAGAGTCTCACCTGAGG |
| MGG_04404-R | GCACGCCGCTCATGTACATGAG |
| MGG_08985-F | GACGCCGGTGTGCGCAAG |
| MGG_08985-R | GGTGTCTGACCAGTGGTGCTG |
| MGG_09218-F | CATCACGGCTAGCGTTGCTG |
| MGG_09218-R | GCTCCACGGCCAGGTTG |
| MGG_09433-F | GCGGCCATGCTCGAGGTG |
| MGG_09433-R | GCAAAGAATTGGGCATCGGCATC |
| MGG_02625-F | CGGTTGCACTTCGGCTGGC |
| MGG_02625-R | CCTCCTTGGATCCACTGGCG |
| MGG_00212-F | CAAGCCTGCTGCCATGGCCAG |
| MGG_00212-R | CCATGATCTCGGCCGAGATGTAG |
| MGG_00423-F | CAACCAGGTCGTGGGCGG |
| MGG_00423-R | CTCCGGCTTGAACGACCGTATC |
| MGG_01993-F | GCAAGCACTGCAATGGCTGC |
| MGG_01993-R | CCGACGACTGGTGCGGTAC |
| MGG_05792-F | GCTTCAGAGCGTCGAGATCCG |
| MGG_05792-R | CAGCCGAGGGCATATCTCCATAC |
| MGG_06326-F | GAGCTGGCCTGTCCGTGG |
| MGG_06326-R | CCGACATACAGGCGTGGCTG |
| MGG_13248-F | GAGGCAACAGAGACGGACAGC |
| MGG_13248-R | CTTCCATCAGGTGGTAGGTAGCTGG |
| MGG_04258-F | CGGCACGTGCTACCCCATG |
| MGG_04258-R | CCACTGGCCACCACTGCTAC |
| MGG_02234-F | CGTGCAACGGCGAATGGAG |
| MGG_02234-R | GATGCAGATCCAGTCGGCGC |
| MGG_16660-F | CTGGCCCAGTCGGCCTTC |
| MGG_16660-R | CGCTCCAACCCTTGCGAC |
