# Supplementary material for: Transcriptomics of the Rice Blast Fungus Magnaporthe oryzae in Response to the Bacterial Antagonist Lysobacter enzymogenes Reveals Candidate Fungal Defense Response Genes
Source: PLoS One. 2013 Oct 3;8(10):e76487. doi: 10.1371/journal.pone.0076487 (PMC3789685; doi:10.1371/journal.pone.0076487)
Supplement: Table S3 — qRT-PCR results for validation of M. oryzae-L. enzymogenes RNA-Seq results. (DOCX) [file pone.0076487.s005.docx]

## Table S3. Real-time qRT-PCR results for validation of *M. oryzae*-*L. enzymogenes* RNA-Seq results.

|  |  | |  | **C3^1^ (3hpi)** | | **DCA^2^ (3hpi)** | | **C3 (9hpi)** | | **DCA (9hpi)** | |
| --- | --- | --- | --- | --- | --- | --- | --- | --- | --- | --- | --- |
|  | **Gene** | | **Description** | **RNA-S^3^** | **qPCR^4^** | **RNA-S** | **qPCR** | **RNA-S** | **qPCR** | **RNA-S** | **qPCR** |
| 1 | MGG_03090 | | Sphingosine N-acyltransferase lag1 | -0.40 | -1.59 | 0.31 | 1.44 | *-0.12 | -1.36 | -0.28 | -1.66 |
| 2 | MGG_05189 | | Sphingosine N-acyltransferase lac1 | -0.34 | -1.45 | -0.30 | -1.11 | 0.24 | -1.01 | -1.03 | -3.34 |
| 3 | MGG_10668 | | Ceramide glucosyltransferase | -0.55 | -1.70 | *-0.05 | 1.00 | -0.51 | -1.32 | -1.36 | -3.79 |
| 4 | MGG_00153 | | Sphingolipid base-responsive protein LSP1 | 0.91 | 1.22 | -0.50 | -1.31 | 0.25 | -1.08 | -0.10 | -1.98 |
| 5 | MGG_05499 | | Serine/threonine protein kinase | -3.19 | -1.85 | 2.67 | 1.60 | -1.50 | -1.69 | -4.09 | -5.38 |
| 6 | MGG_07580 | | Glucose oxidase | *4.56 | 1.20 | -1.00 | 1.18 | 2.55 | 1.66 | 2.36 | 1.63 |
| 7 | MGG_01236 | | WD repeat-containing protein slp1 | -0.87 | -1.82 | 1.06 | 2.42 | -0.82 | -1.02 | -2.28 | -3.89 |
| 8 | MGG_06035 | | FK506-binding protein 1B | -1.58 | -1.73 | 2.89 | 1.73 | -1.73 | -2.13 | -2.99 | -4.97 |
| 9 | MGG_02710 | | Peroxiredoxin type-2 | -3.99 | -3.27 | 8.38 | 2.21 | -4.11 | -2.75 | -5.47 | -5.63 |
| 10 | MGG_01081 | | Peroxin 14/17 | -1.04 | -1.58 | 1.45 | 1.44 | -0.65 | 1.80 | -2.08 | -6.23 |
| 11 | MGG_04404 | | Pisatin demethylase | -2.72 | -1.23 | 5.54 | 1.03 | *0.81 | 1.59 | -2.01 | -1.97 |
| 12 | MGG_08985 | | Beta-xylosidase | -3.03 | -1.23 | 7.60 | 1.91 | -4.50 | -2.80 | -6.12 | -6.63 |
| 13 | MGG_09218 | | Oxidoreductase | -1.04 | -1.47 | 1.26 | 1.91 | *-0.28 | -1.08 | -1.60 | -6.02 |
| 14 | MGG_09433 | | Endoglucanase family 5 glycoside hydrolase | -1.94 | -2.06 | 3.66 | 1.41 | -2.36 | -2.31 | -4.76 | -10.40 |
| 15 | MGG_02625 | | Superoxide dismutase | -1.91 | -1.83 | 1.06 | 1.65 | -1.14 | -1.35 | -1.57 | -2.42 |
| 16 | MGG_00212 | | Superoxide dismutase | -0.90 | -1.71 | -0.78 | 1.02 | -1.51 | -1.77 | -2.89 | -6.98 |
| 17 | MGG_00423 | | Laccase TilA | 16.90 | 1.42 | 5.55 | 2.31 | *-0.16 | -1.47 | *1.90 | -1.36 |
| 18 | MGG_01993 | | Hypothetical protein | 10.04 | 2.67 | 8.89 | 2.32 | 4.09 | 4.75 | 1.57 | 3.31 |
| 19 | MGG_05792 | | Hypothetical protein | -13.03 | -1.03 | 40.48 | -1.80 | -5.18 | -1.55 | -5.25 | -3.51 |
| 20 | MGG_06326 | | Vacuolar ATP synthase proteolipid subunit | -0.74 | -1.52 | 1.07 | 1.84 | 0.83 | 1.68 | -1.00 | -2.50 |
| 21 | MGG_13248 | | Hypothetical protein | 1.00 | -1.19 | 16.42 | -1.57 | *0.00 | -1.33 | 7.80 | -1.19 |
| 22 | MGG_04258 | | Hypothetical protein | 7.42 | 4.17 | *1.14 | 1.50 | 387.73 | 276.92 | 949.46 | 337.79 |
| 23 | MGG_02234 | | Hypothetical protein | -1.00 | 1.70 | -1.00 | 1.18 | 1.43 | 3.11 | 12.39 | 5.67 |
| 24 | MGG_16660 | | Hypothetical protein | *-0.34 | 1.67 | *0.96 | -1.54 | *2.47 | 1.17 | 13.03 | 2.74 |
| Validation (average 90.7%) | |  | | 91.7% | | 87.5% | | 87.5% | | 95.8% | |

^1^*M. oryzae* was challenged with the *L. enzymogenes* wild-type strain C3; fold changes were calculated relative to control (*M. oryzae* in 1x PBS buffer)

^2^*M. oryzae* was challenged with the *L. enzymogenes* mutant strain DCA; fold changes were calculated relative to control (*M. oryzae* in 1x PBS buffer)

^3^RNA-Seq fold changes

^4^real-time qRT-PCR fold changes

* means it is not significant in the RNA-seq considering p-value ≤0.01; cDNA was prepared from the same RNA used for RNA-seq for genes 1 to 9, and with RNA from a second extraction of the same tissue for genes 10 to 24.
