# Supplementary material for: Transcriptomics of the Rice Blast Fungus Magnaporthe oryzae in Response to the Bacterial Antagonist Lysobacter enzymogenes Reveals Candidate Fungal Defense Response Genes
Source: PLoS One. 2013 Oct 3;8(10):e76487. doi: 10.1371/journal.pone.0076487 (PMC3789685; doi:10.1371/journal.pone.0076487)
Supplement: Table S4 — One hundred M. oryzae annotated genes repressed by L. enzymogenes wild-type strain C3 and induced by mutant DCA at 3 hpi. (DOCX) [file pone.0076487.s006.docx]

**Table S4.** One hundred *M. oryzae* annotated genes repressed by *L. enzymogenes* wild-type strain C3 and induced by mutant DCA at 3 hpi.

| **Gene** | **Description** | **C3** | **DCA** |
| --- | --- | --- | --- |
| MGG_12423.6 | Choline monooxygenase | -22.69 | 21.42 |
| MGG_01365.6 | FAD binding domain-containing protein | -15.91 | 37.65 |
| MGG_07219.6 | Conidial yellow pigment biosynthesis polyketide synthase | -13.02 | 5.58 |
| MGG_03773.6 | Hydroxyquinol 1,2-dioxygenase | -12.6 | 25.23 |
| MGG_09355.6 | NACHT domain-containing protein | -11.73 | 22.76 |
| MGG_00993.6 | Vacuolar iron transporter Ccc1 | -9.08 | 4.41 |
| MGG_05989.6 | Seprase | -9.04 | 5.48 |
| MGG_01231.6 | Sorbitol dehydrogenase | -7.40 | 2.46 |
| MGG_03793.6 | 2,3-dihydroxybenzoic acid decarboxylase | -6.66 | 45.29 |
| MGG_03764.6 | Salicylate hydroxylase | -6.37 | 20.72 |
| MGG_08615.6 | Dihydrodipicolinate synthase | -6.27 | 7.40 |
| MGG_05809.6 | Alpha-N-arabinofuranosidase A | -5.65 | 7.13 |
| MGG_17864.6 | Glycosyl hydrolase family 88 | -5.46 | 3.21 |
| MGG_04014.6 | Dihydroxyacetone kinase | -5.34 | 3.21 |
| MGG_07553.6 | CFEM domain-containing protein | -5.34 | 12.85 |
| MGG_00689.6 | Propionate-CoA ligase | -5.28 | 16.96 |
| MGG_14292.6 | Leupeptin-inactivating enzyme 1 | -5.15 | 5.05 |
| MGG_12589.6 | 4-coumarate-CoA ligase 1 | -5.08 | 7.33 |
| MGG_09857.6 | Sorbitol dehydrogenase | -4.84 | 16.37 |
| MGG_06587.6 | Leucyl aminopeptidase | -4.61 | 3.79 |
| MGG_01863.6 | Aminopeptidase Y | -4.48 | 3.50 |
| MGG_04225.6 | MFS quinate transporter | -4.32 | 3.47 |
| MGG_09601.6 | Alpha-xylosidase | -4.19 | 8.41 |
| MGG_02710.6 | Peroxiredoxin type-2 | -3.99 | 8.38 |
| MGG_00220.6 | NADP-dependent alcohol dehydrogenase 6 | -3.93 | 5.89 |
| MGG_04419.6 | Acetylcholinesterase | -3.93 | 8.55 |
| MGG_04839.6 | Peroxisomal dehydratase | -3.91 | 1.57 |
| MGG_13334.6 | General amino acid permease AGP2 | -3.88 | 2.45 |
| MGG_02051.6 | CAMK protein kinase | -3.84 | 2.53 |
| MGG_07502.6 | Chaperone dnaJ 2 | -3.67 | 4.19 |
| MGG_01924.6 | Benzoate 4-monooxygenase cytochrome P450 | -3.64 | 5.17 |
| MGG_02252.6 | Tetrahydroxynaphthalene reductase | -3.58 | 4.00 |
| MGG_07884.6 | Ribose 5-phosphate isomerase | -3.50 | 5.28 |
| MGG_06784.6 | Aldo-keto reductase | -3.32 | 8.15 |
| MGG_02617.6 | 2-methylcitrate synthase | -3.27 | 1.94 |
| MGG_03165.6 | Heat shock protein 60 | -3.21 | 3.05 |
| MGG_05499.6 | Serine/threonine protein kinase | -3.19 | 2.67 |
| MGG_04584.6 | HNRNP arginine N-methyltransferase | -3.08 | 2.06 |
| MGG_08985.6 | Beta-xylosidase | -3.03 | 7.60 |
| MGG_10730.6 | Potassium/sodium efflux P-type ATPase | -3.02 | 4.26 |
| MGG_05383.6 | HHE domain-containing protein | -3.01 | 4.95 |
| MGG_01202.6 | D-lactate dehydrogenase | -2.97 | 3.89 |
| MGG_07250.6 | DEAD/DEAH box helicase | -2.96 | 3.39 |
| MGG_01256.6 | Phosphoribosylaminoimidazole carboxylase | -2.87 | 1.97 |
| MGG_01270.6 | 2,4-dihydroxyhept-2-ene-1,7-dioic acid aldolase | -2.82 | 2.03 |
| MGG_03853.6 | Aldose 1-epimerase | -2.78 | 1.77 |
| MGG_04404.6 | Pisatin demethylase | -2.72 | 5.54 |
| MGG_07935.6 | Galactonate dehydratase | -2.64 | 3.99 |
| MGG_07463.6 | D-galacturonic acid reductase | -2.60 | 1.96 |
| MGG_14744.6 | Phosphotransferase enzyme family domain-containing protein | -2.56 | 3.07 |
| MGG_00652.6 | Salicylaldehyde dehydrogenase | -2.53 | 1.97 |
| MGG_03097.6 | Oxidoreductase | -2.53 | 7.13 |
| MGG_09072.6 | Alcohol oxidase | -2.50 | 9.88 |
| MGG_10662.6 | Alpha-glucosidase | -2.42 | 2.66 |
| MGG_03921.6 | 3-oxoacyl-[acyl-carrier-protein] reductase | -2.36 | 2.77 |
| MGG_11519.6 | Alcohol dehydrogenase | -2.28 | 3.56 |
| MGG_03095.6 | Dihydroxyacetone kinase | -2.19 | 6.5 |
| MGG_15357.6 | MYB DNA-binding domain-containing protein | -2.17 | 2.99 |
| MGG_05025.6 | Fatty acid transporter | -2.16 | 2.28 |
| MGG_09867.6 | N-acetyltransferase ats1 | -2.13 | 1.65 |
| MGG_07782.6 | Dehydroquinase class II | -2.11 | 6.19 |
| MGG_01742.6 | Elongation factor 2 | -2.09 | 1.98 |
| MGG_05433.6 | Solute carrier family 6 protein | -2.08 | 1.56 |
| MGG_04710.6 | Translational activator GCN1 | -2.07 | 1.52 |
| MGG_06332.6 | Peroxisomal adenine nucleotide transporter 1 | -2.01 | 3.37 |
| MGG_03774.6 | CAIB/BAIF family enzyme | -2.00 | 2.81 |
| MGG_08623.6 | Neutral alpha-glucosidase AB | -1.96 | 1.75 |
| MGG_09433.6 | Endoglucanase family 5 glycoside hydrolase | -1.94 | 3.66 |
| MGG_03414.6 | Short chain dehydrogenase/reductase family protein | -1.92 | 2.25 |
| MGG_06367.6 | Vesicular integral-membrane protein VIP36 | -1.92 | 1.56 |
| MGG_04938.6 | C-3 sterol dehydrogenase/C-4 decarboxylase | -1.89 | 1.60 |
| MGG_08778.6 | Aromatic ring-opening dioxygenase | -1.89 | 2.62 |
| MGG_15250.6 | Quinic acid-X | -1.88 | 1.55 |
| MGG_08980.6 | Stress-induced-phosphoprotein 1 | -1.87 | 1.51 |
| MGG_10357.6 | Prolyl-tRNA synthetase | -1.85 | 2.54 |
| MGG_02016.6 | Serine/threonine protein kinase | -1.82 | 3.14 |
| MGG_03900.6 | Aldehyde dehydrogenase | -1.82 | 3.14 |
| MGG_08163.6 | 30S ribosomal protein S14p/S29e | -1.82 | 1.54 |
| MGG_05889.6 | Lactose permease | -1.80 | 3.97 |
| MGG_04550.6 | Calcium-translocating P-type ATPase | -1.78 | 2.96 |
| MGG_00359.6 | Delta(3,5)-Delta(2,4)-dienoyl-CoA isomerase | -1.77 | 1.66 |
| MGG_08622.6 | Nucleoside diphosphate kinase | -1.77 | 2.99 |
| MGG_13230.6 | Oxidoreductase | -1.77 | 6.10 |
| MGG_09194.6 | 60S ribosomal protein L17 | -1.75 | 1.57 |
| MGG_07877.6 | Dipeptidyl-peptidase V | -1.71 | 2.05 |
| MGG_11754.6 | ABC transporter SMDR1 | -1.71 | 4.86 |
| MGG_03510.6 | UDP-glucose:glycoprotein glucosyltransferase | -1.68 | 3.69 |
| MGG_06860.6 | Coatomer subunit beta | -1.65 | 1.58 |
| MGG_09404.6 | Feruloyl esterase B | -1.65 | 2.84 |
| MGG_03094.6 | Triosephosphate isomerase | -1.63 | 4.31 |
| MGG_04385.6 | Urea active transporter | -1.63 | 1.58 |
| MGG_09175.6 | Beta-lactamase | -1.63 | 4.09 |
| MGG_04467.6 | 60S acidic ribosomal protein P0 | -1.62 | 2.30 |
| MGG_03201.6 | Acetyl-coenzyme A synthetase | -1.61 | 4.11 |
| MGG_05503.6 | High-affinity nickel-transporter nixA | -1.60 | 1.69 |
| MGG_01387.6 | Endonuclease/exonuclease/phosphatase | -1.58 | 1.87 |
| MGG_03016.6 | 3-oxoacyl-[acyl-carrier-protein] reductase | -1.58 | 2.03 |
| MGG_06035.6 | FK506-binding protein 1B | -1.58 | 2.89 |
| MGG_07048.6 | 60S ribosomal protein L5 | -1.57 | 1.93 |
| MGG_01637.6 | Phosphomannomutase | -1.50 | 2.54 |
