# Supplementary material for: Transcriptomics of the Rice Blast Fungus Magnaporthe oryzae in Response to the Bacterial Antagonist Lysobacter enzymogenes Reveals Candidate Fungal Defense Response Genes
Source: PLoS One. 2013 Oct 3;8(10):e76487. doi: 10.1371/journal.pone.0076487 (PMC3789685; doi:10.1371/journal.pone.0076487)
Supplement: Table S5 — Results of qRT-PCR performed on M. oryzae mycelial samples challenged with L. enzymogenes. M. oryzae mycelia (no spores) were challenged with L. enzymogenes wild-type strain C3 and mutant DCA and the expression level of nineteen genes was examined by qRT-PCR and compared to the RNA-seq results. (DOCX) [file pone.0076487.s007.docx]

## Table S5. Results of qRT-PCR performed on *M. oryzae* mycelial samples challenged with *L. enzymogenes*. *M. oryzae* mycelia (no spores) was challenged with *L. enzymogenes* wild-type strain C3 and mutant DCA and the expression level of nineteen genes was examined by qRT-PCR and compared to the RNA-seq results.

|  |  | | **C3** | | | **DCA** | |
| --- | --- | --- | --- | --- | --- | --- | --- |
| **Gene** | **Description** | | **RNA-S** | **qPCR** | | **RNA-S** | **qPCR** |
| MGG_07219.6 | Conidial yellow pigment biosynthesis polyketide synthase | | -13.02 | -1.71 | | 5.58 | 2.00 |
| MGG_10668.6 | Ceramide glucosyltransferase (ceramide synthase) | | -0.55 | -1.46 | | -0.05 | 1.36 |
| MGG_03090.6 | Sphingosine N-acyltransferase lag1 | | -0.40 | -1.40 | | 1.44 | 1.06 |
| MGG_01081.6 | Peroxin 14/17 | | -1.04 | -1.30 | | 1.45 | 1.05 |
| MGG_00153.6 | Sphingolipid long chain base-responsive protein LSP1 | | 0.91 | -1.21 | | -0.50 | 1.04 |
| MGG_05499.6 | Serine/threonine protein kinase | | -3.19 | -1.13 | | 2.67 | 1.56 |
| MGG_10730.6 | Potassium/sodium efflux P-type ATPase | | -3.02 | -1.12 | | 4.26 | 3.94 |
| MGG_02710.6 | Peroxiredoxin type-2 | | -3.99 | -1.11 | | 8.38 | 1.00 |
| MGG_06035.6 | FK506-binding protein 1B | | -1.58 | -1.03 | | 2.89 | 1.10 |
| MGG_07580.6 | Glucose oxidase | | 4.56 | 1.21 | | -1.00 | -1.16 |
| MGG_06326.6 | Vacuolar ATP synthase proteolipid subunit | | -0.74 | 1.47 | | 1.07 | -1.15 |
| MGG_03793.6 | 2,3-dihydroxybenzoic acid decarboxylase | | -6.66 | 1.12 | | 45.29 | 2.23 |
| MGG_02625.6 | Superoxide dismutase | | -1.91 | 1.48 | | 1.06 | 1.83 |
| MGG_09433.6 | Endoglucanase family 5 glycoside hydrolase | | -1.94 | 1.13 | | 3.66 | 1.83 |
| MGG_03165.6 | Heat shock protein 60 | | -3.21 | 1.82 | | 3.05 | 1.21 |
| MGG_04550.6 | Calcium-translocating P-type ATPase | | -1.78 | -1.17 | | 2.96 | -1.15 |
| MGG_02051.6 | CAMK protein kinase | | -3.84 | -1.26 | | 2.53 | -1.30 |
| MGG_01236.6 | WD repeat-containing protein slp1 | | -0.87 | -2.57 | | 1.06 | -1.84 |
| MGG_08985.6 | Beta-xylosidase | | -3.03 | -3.96 | | 7.60 | -2.93 |
| Validation (average 65.9%) | | 68.5% | | | 63.2% | | |
