# Supplementary material for: Transcriptomics of the Rice Blast Fungus Magnaporthe oryzae in Response to the Bacterial Antagonist Lysobacter enzymogenes Reveals Candidate Fungal Defense Response Genes
Source: PLoS One. 2013 Oct 3;8(10):e76487. doi: 10.1371/journal.pone.0076487 (PMC3789685; doi:10.1371/journal.pone.0076487)
Supplement: Table S7 — Thirty-three genes that contain the promoter element for AZF1 from S. cerevisiae. (DOCX) [file pone.0076487.s009.docx]

## Table S5. Thirty-three genes that contain the promoter element for AZF1 from *S. cerevisiae*.

| **Gene** | **Description** | **AZF1 element^1^** |
| --- | --- | --- |
| MGG_07553.6 | CFEM domain-containing protein | 4 |
| MGG_04225.6 | MFS quinate transporter | 4 |
| MGG_09194.6 | 60S ribosomal protein L17 | 3 |
| MGG_01256.6 | Phosphoribosylaminoimidazole carboxylase | 2 |
| MGG_07463.6 | D-galacturonic acid reductase | 2 |
| MGG_04385.6 | Urea active transporter | 2 |
| MGG_05503.6 | High-affinity nickel-transporter nixA | 2 |
| MGG_07219.6 | Conidial yellow pigment biosynthesis polyketide synthase | 1 |
| MGG_03773.6 | Hydroxyquinol 1,2-dioxygenase | 1 |
| MGG_09355.6 | NACHT domain-containing protein | 1 |
| MGG_05809.6 | Alpha-N-arabinofuranosidase A | 1 |
| MGG_04014.6 | Dihydroxyacetone kinase | 1 |
| MGG_00689.6 | Propionate-CoA ligase | 1 |
| MGG_14292.6 | Leupeptin-inactivating enzyme 1 | 1 |
| MGG_06784.6 | Aldo-keto reductase | 1 |
| MGG_05499.6 | Serine/threonine protein kinase | 1 |
| MGG_08985.6 | Beta-xylosidase | 1 |
| MGG_07250.6 | DEAD/DEAH box helicase | 1 |
| MGG_07935.6 | Galactonate dehydratase | 1 |
| MGG_03097.6 | Oxidoreductase | 1 |
| MGG_03921.6 | 3-oxoacyl-[acyl-carrier-protein] reductase | 1 |
| MGG_05433.6 | Solute carrier family 6 protein | 1 |
| MGG_06332.6 | Peroxisomal adenine nucleotide transporter 1 | 1 |
| MGG_09433.6 | Endoglucanase family 5 glycoside hydrolase | 1 |
| MGG_15250.6 | Quinic acid-X | 1 |
| MGG_03900.6 | Aldehyde dehydrogenase | 1 |
| MGG_05889.6 | Lactose permease | 1 |
| MGG_04550.6 | Calcium-translocating P-type ATPase | 1 |
| MGG_11754.6 | ABC transporter SMDR1 | 1 |
| MGG_03094.6 | Triosephosphate isomerase | 1 |
| MGG_04467.6 | 60S acidic ribosomal protein P0 | 1 |
| MGG_01387.6 | Endonuclease/exonuclease/phosphatase | 1 |
| MGG_03016.6 | 3-oxoacyl-[acyl-carrier-protein] reductase | 1 |

^1^ number of times element appears in promoter
