# Supplementary material for: Transcriptomics of the Rice Blast Fungus Magnaporthe oryzae in Response to the Bacterial Antagonist Lysobacter enzymogenes Reveals Candidate Fungal Defense Response Genes
Source: PLoS One. 2013 Oct 3;8(10):e76487. doi: 10.1371/journal.pone.0076487 (PMC3789685; doi:10.1371/journal.pone.0076487)
Supplement: Table S8 — Twenty-three genes that contain the promoter element for STP2 from S. cerevisiae. (DOCX) [file pone.0076487.s010.docx]

**Table S6.** Twenty-three genes that contain the promoter element for STP2 from *S. cerevisiae*.

| **Gene** | **Description** | **STP2 element** |
| --- | --- | --- |
| MGG_07782.6 | Dehydroquinase class II | 6 |
| MGG_09072.6 | Alcohol oxidase | 5 |
| MGG_04710.6 | Translational activator GCN1 | 5 |
| MGG_09601.6 | Alpha-xylosidase | 3 |
| MGG_01202.6 | D-lactate dehydrogenase | 3 |
| MGG_13334.6 | General amino acid permease AGP2 | 2 |
| MGG_01924.6 | Benzoate 4-monooxygenase cytochrome P450 | 2 |
| MGG_04550.6 | Calcium-translocating P-type ATPase | 2 |
| MGG_00993.6 | Vacuolar iron transporter Ccc1 | 1 |
| MGG_05989.6 | Seprase | 1 |
| MGG_03764.6 | Salicylate hydroxylase | 1 |
| MGG_12589.6 | 4-coumarate-CoA ligase 1 | 1 |
| MGG_09857.6 | Sorbitol dehydrogenase | 1 |
| MGG_00220.6 | NADP-dependent alcohol dehydrogenase 6 | 1 |
| MGG_07884.6 | Ribose 5-phosphate isomerase | 1 |
| MGG_06784.6 | Aldo-keto reductase | 1 |
| MGG_10662.6 | Alpha-glucosidase | 1 |
| MGG_03095.6 | Dihydroxyacetone kinase | 1 |
| MGG_02016.6 | Serine/threonine protein kinase | 1 |
| MGG_03900.6 | Aldehyde dehydrogenase | 1 |
| MGG_05889.6 | Lactose permease | 1 |
| MGG_06860.6 | Coatomer subunit beta | 1 |
| MGG_05503.6 | High-affinity nickel-transporter nixA | 1 |
